# Comparative genomics analysis provides insights into evolution and stress responses of *Lhcb* genes in Rosaceae fruit crops

**DOI:** 10.1186/s12870-023-04438-x

**Published:** 2023-10-11

**Authors:** Xiaolong Li, Zeyu Jiang, Chaofan Zhang, Kefan Cai, Hui Wang, Weiyi Pan, Xuepeng Sun, Yongbin Gao, Kai Xu

**Affiliations:** 1 Key Laboratory of Quality and Safety Control for Subtropical Fruit and Vegetable, Ministry of Agriculture and Rural Affairs, College of Horticulture Science, Zhejiang A&F University, Hangzhou, 311300 Zhejiang China; 2 College of Agriculture and Biotechnology, Zhejiang University, Hangzhou, 310058 Zhejiang China

**Keywords:** Rosaceae, Stress responses, Evolution

## Abstract

**Background:**

Light-harvesting chlorophyll a/b binding proteins (*Lhcb*) play crucial roles in plant growth, development, and the response to abiotic stress in higher plants. Previous studies have reported that *Lhcb* genes were involved in the phytochrome regulation and responded to different light and temperature conditions in Poaceae (such as maize). However, the evolution and functions of *Lhcb* genes remains poorly characterized in important Rosaceae species.

**Results:**

In this investigation, we conducted a genome-wide analysis and identified a total of 212 *Lhcb* genes across nine Rosaceae species. Specifically, we found 23 *Lhcb* genes in *Fragaria vesca*, 20 in *Prunus armeniaca*, 33 in *Malus domestica ‘Gala’*, 21 in *Prunus persica*, 33 in *Rosa chinensis*, 29 in *Pyrus bretschneideri*, 18 in *Rubus occidentalis*, 20 in *Prunus mume*, and 15 in *Prunus salicina*. Phylogenetic analysis revealed that the *Lhcb* gene family could be classified into seven major subfamilies, with members of each subfamily sharing similar conserved motifs. And, the functions of each subfamily was predicted based on the previous reports from other species. The Lhcb proteins were highly conserved within their respective subfamilies, suggesting similar functions. Interestingly, we observed similar peaks in *Ks* values (0.1–0.2) for *Lhcb* genes in apple and pear, indicating a recent whole genome duplication event (about 30 to 45 million years ago). Additionally, a few *Lhcb* genes underwent tandem duplication and were located across all chromosomes of nine species of Rosaceae. Furthermore, the analysis of the *cis*-acting elements in the 2000 bp promoter region upstream of the pear *Lhcb* gene revealed four main categories: light response correlation, stress response correlation, hormone response correlation, and plant growth. Quantitative expression analysis demonstrated that *Lhcb* genes exhibited tissue-specific expression patterns and responded differently to low-temperature stress in Rosaceae species.

**Conclusions:**

These findings shed light on the evolution and phylogeny of *Lhcb* genes in Rosaceae and highlight the critical role of Lhcb in pear’s response to low temperatures. The results obtained provide valuable insights for further investigations into the functions of *Lhcb* genes in Rosaceae, and these functional genes will be used for further fruit tree breeding and improvement to cope with the current climate changes.

**Supplementary Information:**

The online version contains supplementary material available at 10.1186/s12870-023-04438-x.

## Introduction

Green plants possess the ability to convert light energy into chemical energy through photosynthesis, fueling essential cellular processes. Pear trees, for instance, rely on leaves for photosynthesis to generate energy after undergoing flowering, pollination, and fertilization. Chlorophyll, a pigment involved in photosynthesis, captures and transfers light energy. Within the photosystem II (PSII) complex, there are various components, including the peripheral light trapping (antenna) pigment-protein complex (LhcII), internal antenna pigment-protein complex (CP43 and CP47) [1, 2], reaction center pigment-protein complex (PSII-RC), peripheral proteins such as 33 kDa and 17 kDa. The effective functioning of PSII depends on its ability to absorb light energy, a role fulfilled by Lhc proteins that serve as light traps during photosynthesis. The Lhc superfamily, exclusive to plants, comprises four subfamilies: chlorophyll-a/b-binding proteins (Lhc), light-harvesting-like (Lil), photosystem II subunit S (PsbS), and ferrochelatase II (FCII). The Lhc subfamily can be further classified into two groups, namely Lhca and Lhcb. The chlorophyll a/b binding domain (PF00504) is prevalent a member of the Lhc superfamily across various plant species. To date, Lhc superfamily members have been discovered in various plants, including *Arabidopsis* [3], rice (*Oryza sativa*) [4, 5], kiwifruit (*A. chinensis* and *A. eriantha*) [6], tomato (*Lycopersicon esculentum*) [7, 8], and apple (*Malus domestica*) [9].

Apart from their light-capturing role, members of the Lhc family also contribute to the regulation of plant growth and development. For instance, the *Lhcb* gene in *Arabidopsis* had primarily a hand in seed germination and post-germination growth With regards to the plant hormone abscisic acid (ABA). The down-regulation of the *AtLhcb1* gene in *Arabidopsis* resulted in slightly smaller leaves, lighter colors, and lowered chlorophyll content compared to the wild type [10]. In celery (*Apium graveolens* L), the up-regulation of the *AgLhcb1* gene increased efficiency of photosynthetic, making it a potential reference for calculating photosynthetic rates [11]. Overexpression of the *SaLhcb2* gene in *Sedum alfredii* led to increased shoot and root biomass [12]. In *Hordeum vulgare L.*, five single nucleotide polymorphisms (SNPs) in the *Lhcb1* gene were observably link with various agronomic traits, including plant height, ear length, grains per ear, thousand-grain weight, flag leaf area, and leaf color [13]. Similarly, overexpression of the *MdLhcb4.3* gene in apple increased chlorophyll content in *Arabidopsis*, while knockout mutants of the *AtLhcb6*, *AtLhcb5*, and *AtLhcb4* genes showed significantly lower chlorophyll content in *Arabidopsis* [9]*.* Moreover, the Lhc family was crucial for plant stress response and stress resistance. In *Apium graveolens*, the expression of *Lhcb1* was up-regulated under cold, heat, salt, and drought stress conditions [11]. In *Arabidopsis*, the Lhcb1-6 genes respond to stomatal movement and participate in ABA signaling, influencing reactive oxygen species (ROS) homeostasis and contributing to plant stress resistance [14, 15]. Overexpression of the *MdLhcb4.3* gene in transgenic *Arabidopsis* and apple callus enhanced their tolerance to drought and osmotic stress. In tobacco, overexpression of the *LeLhcb2* gene improved tolerance to low-temperature stress and reduced photo-oxidation of PSII [7]. Formaldehyde stress impacted the expression of photosynthetic genes *Lhcb2.1* and *Lhcb3* in *Arabidopsis*.

The Rosaceae family encompasses a diverse range of fruit trees and ornamental flowers, playing a vital role in our daily lives. However, the flowering patterns of these plants are being affected by global climate change. One meteorological phenomenon known as "inverted spring cold" poses a significant threat by damaging fully developed flower buds and disrupting pollinator activity. Flowers and well-formed buds are particularly susceptible to low temperatures, leading to reduced fruit-setting rates and substantial agricultural losses [16]. Previous studies have highlighted the crucial role of the *Lhcb* gene in *Arabidopsis*’s adaptation to low temperatures. However, limited information is available regarding the *Lhcb* gene family in Rosaceae. In this study, we aim to address this gap by identifying and characterizing members of the *Lhcb* gene family in nine Rosaceae species, including strawberry (*Fragaria vesca*), pear (*Pyrus bretschneideri*), apple (*Malus domestica*), peach (*Pyrus bretschneideri*), rose (*Rosa chinensis*), black raspberry (*Rubus occidentalis*), Japanese apricot (*Prunus mume*), and Japanese Plum (*Prunus salicina*). We conducted a comprehensive analysis of phylogeny, gene duplication, chromosome localization, and collinearity, promoter motif, and selection analysis. Additionally, we examined the expression profiles of *Lhcb* genes in various tissues of multiple Rosaceae species, along with their responses to low-temperature stress. Our findings will serve as a valuable reference for understanding the evolutionary relationships and biological functions of the *Lhcb* gene family in Rosaceae.

## Materials and methods

### Recognition of representatives of the *Lhcb* gene family

Genome sequences and annotations for nine Rosaceae species were retrieved from the Rosaceae Genome Database (GDR: https://www.rosaceae.org/). We employed the representative genomes as standards: *Prunus persica* ‘Zhongyoutao’ 14 Genome v1.0; *Fragaria vesca* Genome v4.0.a1; *Malus x domestica* Gala haploid v1.0 genome; *Rosa chinensis* Old Blush homozygous Genome v2.0; *Prunus armeniaca* Marouch n14 Whole Genome v1.0; *Rubus occidentalis* whole genome assembly v3.0; *Prunus mume* Tortuosa Genome v1.0; *Prunus salicina* Zhongli No.6 Genome v1.0; *Pyrus bretschneideri* 'DangshanSuli' Genome Assembly v1.1. Complete Lhcb protein alignments of *Arabidopsis* were obtained from The Arabidopsis Information Resource (TAIR10: http://www.Arabidopsis.org/) and utilized for BLASTP searches targeting the protein sequences of nine Rosaceae species possessing an e-value of 1e-10. In addition, a Hidden Markov Model search (HMMsearch) was employed to determine *Lhcb* members according to their respective HMM profile (PF00504) conserved domain. The intersection of genes acquired using these two methods was used as a screening criterion for candidate *Lhcb* genes. Each candidate gene was input to Pfam (http://pfam.xfam.org/) to establish the existence of Lhcb domains. Moreover, the appearance of a chlorophyll A/B binding domain across candidate proteins was established and determined using the Pfam program [17]. The integrity of the domain was confirmed using CDD-search and interpro software. Syntenic blocks were identified using MCScanX software [18], and whole genome duplication (WGD) occurrences were detected upon gene duplications situated on syntenic blocks on duplicated chromosomes [19, 20].

### Construction of a phylogenetic tree

A phylogenetic tree was constructed utilizing the entire amino acid sequences of Lhcb proteins spanning nine Rosaceae species. The sequence alignment of Lhcb proteins was conducted using MUSCLE software (https://www.ebi.ac.uk/Tools/msa/muscle/), with standard settings in MEGA 11 (http://megasoftware.net). The phylogeny was generated through the use of a Neighbor–Joining (NJ) algorithm in MEGA11, and confirmed utilizing a maximum likelihood method (ML) with 1000 bootstrapping repetitions. The final tree topology was presented using itol (https://itol.embl.de/).

### Gene organization, motif attributes, and exploration of *cis*-regulatory elements

The arrangement of genes in the *Lhcb* family was analyzed using the Gene Structure Display Server (GSDS 2.0, http://gsds.cbi.pku.edu.cn/). By employing MEME (http://meme-suite.org/tools/meme) [21], a total of 15 conserved motifs were discerned within Lhcb proteins. Additionally, the PlantCARE databank (https://bioinformatics.psb.ugent.be/webtools/plantcare/html/) was utilized to project *cis*-regulatory elements within the proximal 2000 bp upstream of *Lhcb* genes.

### Chromosomal position, gene copying, and synteny evaluation

TBtools [22] was employed to extract the locations of *Lhcb* genes from the corresponding GFF file. MapChart software (https://www.mapchart.net/) was used to visualize specific chromosome genes. Thereafter, MCScanX software was utilized for the identification of duplication configuration of Lhcb using default settings. The synonymous (Ks) and nonsynonymous (Ka) mutation levels of the replicated *Lhcb* gene pairs were determined utilizing the TBtools software package. The Ks value is often used as a molecular timer to compute the duration since gene replication [23]. Nonsynonymous substitutions (Ka), Ks, and Ka/Ks were computed across six Rosaceae species with MEGA7.0. The Ka/Ks ratio served as a pivotal gauge for assessing the selective pressure on protein-coding genes. A Ka/Ks ratio surpassing 1 indicated the presence of positive selective pressure driving gene evolution and overall advantageous variability. A Ka/Ks ratio of precisely 1 denoted neutral selection, while genes with Ka/Ks ratios below 1 displayed purifying selection [24]. Additionally, the ClustalW 2.0 tool [25] was used to build nucleotide alignments of CDSs across gene families within the nine species, employing corresponding protein sequences. For the analysis of corresponding CDSs, the Jukes-Cantor approach was applied using pairwise deletion. The Ks values for nine Rosaceae species were visualized using the ‘ggplot2’ R package. The syntenic blocks of *Lhcb* were generated through MCScanX software using default settings, and *Lhcb* gene sets were detected using TBtools.

### Transcriptome data investigation

Transcriptome data from 16 diverse strawberry tissues were obtained from strawberry genome resources (http://bioinformatics.towson.edu/strawberry/Default.aspx) [26, 27] as well as the Genome Database for Rosaceae (GDR: https://www.rosaceae.org/) (Fv.2.0a1). This data encompassed carpels, anther, cortex, embryo, leaf, ovule, pulp, bud, seedling, style, wall, microspore, flower, perianth, and receptacle [28, 29]. The RNA-seq data were compiled through the use of the Illumina Hiseq2000 and HiSeq4000 platforms. Clean reads were mapped to the *F. vesca* Genome v2.0.a1 [30] employing Bowtie2, and the gene expression was standardized as RPKM (reads per KB per million) values. Transcriptome data from seven different pear tissues, including buds, stems, ovaries, leaves, petals, sepals, and fruits, were accessed using the PearEXP databank (http://www.peardb.org.cn/). These raw reads are available at the National NCBI under the study of project accession PRJNA498777 [31]. The expression levels of *Lhcb* gene family members from strawberry and pear were obtained from the corresponding expression data and presented utilizing the ‘pheatmap’ R package.

To understand the response mode of the *Lhcb* gene family to low temperature, we downloaded the RNA-seq data of buds under low-temperature stress at different periods and three different tissues (PRJNA577143) of *Prunus armeniaca* [32] from the Sequence Read Archive (SRA: SAMN12791244 to SAMN12791303). The expression data of leaves and corolla under low-temperature environment from woodland strawberry (PRJNA700642) using Gene Expression Omnibus (GEO, https://www.ncbi.nlm.nih.gov/geo/) [33]. The base levels of reads from each gene across apricot and strawberry following read mapping were deposited into NCBI GEO with the accession numbers GSE138792 and GSE166374, respectively. RNA extraction from floral buds of five apricot genotypic representatives was conducted, followed by sequencing using an Illumina NextSeq 500. HISAT2 [34] was utilized to align the clean apricot RNA-seq reads to the reference genome “Marouch n14” of *Prunus armeniaca* OF[35]. The aligned reads were examined through HTSeq-count [36].

### Phenotypic evalution and qPCR assay

Nine rootstock variety 'Douli' pear seedlings with good growth and similar growth state were selected from the greenhouse in Zhejiang Agriculture and Forestry University. Then, each three seedlings were incubated in an artificial climate chamber at different temperatures (4℃, 20℃, 30℃) for treatment. In addition, a pear seedling with similar growth was selected as CK (untreated control) group in the greenhouse and sampled simultaneously with the treatment groups. We amassed juvenile pear leaves over 5 durations: 0 h, 12 h, 24 h, 3 days, and 5 days. Three leaves were collected at each time period as biological replicates, and immediately flash-frozen in liquid nitrogen. The chlorophyll index of pear leaf from different heights of the pear plants was measured using the SPAD-502 Plus (Konica Minolta), and used the boxplot to presented the results. For qPCR assays, the entire RNA complement was obtained from samples and exposed to DNase for genomic DNA removal. This was followed by reverse transcription to synthesize the first cDNA strand. qPCR was conducted using a SYBR reaction mix. Tubulin was employed as an internal reference. Relative expression of the examined genes were determined through the use of biological triplicates, and expression was computed utilizing the 2^−ΔΔCT^ method. qPCR primers outlined in Additional file and Table S4 were engineered to increase the candidate gene sequence signals using NCBI web services (National Center for Biotechnology Information, https://www.ncbi.nlm.nih.gov/tools/primerblast/), with 55–60℃ of T_m_ value and 40%-60% of GC content.

## Results

### Identification of *Lhcb* genes in nine species of Roseaceae

**Fig. 1:**
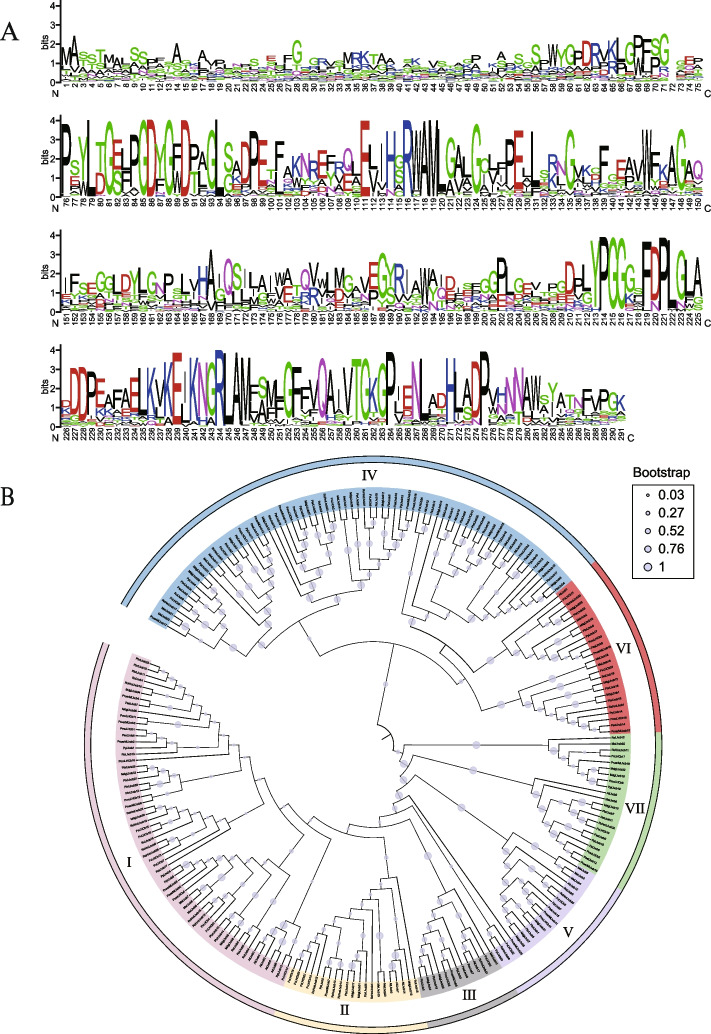
**A** Sequence logos of Lhcb repeats are generated based on the full-length alignments of all Lhcb domains in nine roseceae species: *Malus domestica, Prunus persica, Pyrus bretschneider, Prunus salicina, Prunus mume, Prunus armeniaca, Rosa chinensis, Fragaria vesca* and *Rubus occidentalis.* **B** The phylogenetic tree of *Lhcb* genes from 9 Rosaceae species was clustered according to the classification of *Lhcb* gene in Arabidopsis. Different colored backgrounds represents different clusters. The blue circles represents bootstrap values

BLAST and hmmer methods were used to identify *Lhcb* homologous genes. Based on these methods, 212 complete Lhcb protein sequences were identified from nine species, including 23 in strawberry (*Fragaria vesca*), 33 in apple (*Malus domestica*), 21 in peach (*Prunus persica*), 29 in white pear (*Pyrus bretschneideri*), 33 in Chinese rosa (*Rosa chinensis*), 18 in black raspberry (*Rubus occidentalis*), 15 in Japanese Plum (*Prunus salicina*), 20 in apricot (*Prunus armeniaca*), and 20 in Japanese apricot (*Prunus mume*) (Table 1). Among them, *Fragaria vesca* (23), *Prunus mume* (20), *Prunus persica* (21), and *Prunus armeniaca* (20) had the same number of *Lhcb* genes. In addition, across the 9 species studied, strawberry (0.80‰) had the highest proportion of *Lhcb* genes, while plum (0.53‰) had the lowest. The pear and peach *Lhcb* genes were used as examples for multiple sequence alignment. Our findings indicated that *Lhcb* genes in both pears and peaches contained a chlorophyll a/b binding protein domain (Fig. S1A). We next investigated homologous domain sequence characteristics through multiple alignment analysis using 283 homologous domain amino acid sequences for Lhcb repeats. We obtained the frequencies of the most common amino acids for each location across the Lhcb domain of nine Rosaceae representatives. The height of each letter in the sequence logo is proportional to the occurrence frequency of the corresponding base at that location, represented in bits. The letters in each position are arranged from most conserved to least conserved (Fig. 1A). Our findings suggested that the basic region of the Lhcb domain consisted of 281 basic residues (including the junction), with a few deletions or insertions. In the Rosaceae Lhcb family, Lhcb repeats consist of characteristic amino acids, including a series of evenly distributed and highly conserved proline, glycine, and glutamic acid residues, indicating high amino acid conservation in the Lhcb domain between species in the Rosaceae family. Among these highly conserved residues, some amino acids were changed less frequently. Through comparison to the *Arabidopsis Lhcb* gene family (Fig. S1B), we found that the amino acid distribution in the Lhcb domain of Rosaceae was nearly identical to *Arabidopsis*. Finally, we renamed the Lhcb gene according to the positional order on the chromosome (Table S1).

**Table 1:** Numbers of *Lhcb* genes in nine Rosaceae species

Species name	Chromosome number	Release version	Genome gene number	Identified Lhcb genes	Proportion of Lhcb genes (‰)
*F. vesca*	7	GDR, v4.0a1	28,588	23	0.80
*P. armeniaca*	8	GDR,	27,643	20	0.72
*M. domestica*	17	GDR, v2	45,352	33	0.72
*P. persica*	8	GDR, v1	30,181	21	0.69
*R. chinensis*	7	GDR, v2.0a1	50,387	33	0.65
*P. bretschneider*	17	GDR, v1.1	42,180	29	0.69
*R. occidentalis*	7	GDR, v3.0	33,253	18	0.54
*P. mume*	8	GDR	37,521	20	0.53
*P. salicina*	8	GDR, v2.0	24,448	15	0.61

The version of the Rosaceae species genome data was downloaded from the data sources GDR: Genome Database for Rosaceae (https://www.rosaceae.org/); The proportion of *Lhcb* genes is relative to their whole genome genes in nine Rosaceae species, respectively

### Phylogenetic tree and conserved motif of the Lhcb genes

**Fig. 2:**
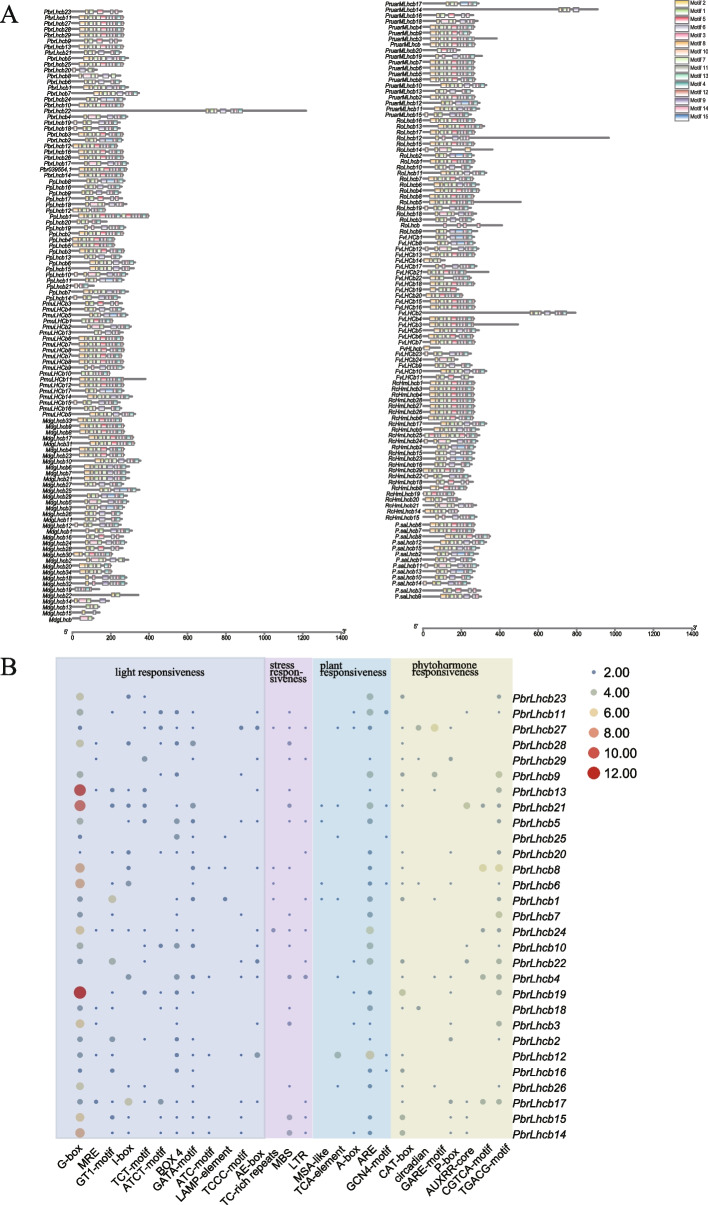
**A** Architecture of conserved protein motifs in *Lhcb* genes from nine Rosaceae species. **B** *PbrLhcb* upstream 2000 bp diagram of different functional *cis*-regulation elements

To investigate the evolutionary relationships within the *Lhcb* gene family, a joint phylogenetic analysis was conducted on the Lhcb proteins from across nine Rosaceae species (212 genes), *Arabidopsis* (17 genes), and cassava (23 genes) using an Neighbor–Joining (NJ) method in MEGA11. To confirm the reliability of the results, bootstrapping was used for 1000 repeats, and the maximum likelihood method (ML) was also used for additional verification. In our generated phylogenetic trees, we uncovered members of the *Arabidopsis Lhcb* gene family on each branch, indicating that gene expansion of the *Lhcb* family occurred prior to the origin of dicotyledonous plants. Consistent with this classification of Lhcb proteins in *Arabidopsis thaliana* and *Manihot esculenta*, all Lhcb proteins were classified into seven distinct subfamilies, namely LhcbI, LhcbII, LhcbIII, LhcbIV, LhcbV, LhcbVI, and LhcbVII (Fig. 1B). Based on cluster analysis, we can conclude that the LhcbIII, LhcbV, and LhcbVII subfamilies are relatively conserved. Using our evolutionary tree, it can be determined that LhcbV and LhcbVII are encoded by a single gene, while Lhcb4 is encoded by several highly conserved genes. To uncover structural changes and possible functional divergences, the coding sequences for the Lhcb genes from the nine Rosaceae species were analyzed using MEME software, identifying a total of 15 conserved motifs. Nearly all gene members within the same clade have several motifs, indicating that the protein is conserved and may have similar functions. The conserved motifs may be involved in transcriptional regulation (Fig. 2A). The LhcbIV subfamily contained the highest number of motifs compared to other subfamilies. Motif 1 was widely distributed across all Lhcb proteins and located in the conserved Chlorophyll a/b binding domain (Fig. S2). Several specific motifs were found only in particular subfamilies. For example, motif 14 and motif 15 were only present in the LhcbIV subfamily, suggesting that genes in LhcbIV have specific functions. To further explore the role of *Lhcb* genes in plant growth, we performed functional prediction for each subfamily based on previous reports (Table S6). The results indicated that LhcbI has a vital role in regulating circadian rhythm, and LhcbII, LhcbIV, and LhcbV respond to stress. At the same time, LhcbIII is closely related to chloroplast biosynthesis, and LhcbVI and LhcbVII influence plant growth and development.

### Upstream 2000 bp *cis*-regulatory element analysis of *PbrLhcb*

*Cis*-acting elements, including promoters, are crucial for regulating transcription and gene expression. The upstream 2000 bp promoter sequences of the pear *Lhcb* gene family members were isolated using TBtools (Fig. 2B). PlantCARE analysis indicated the presence of 29 high-frequency *cis*-acting elements in the pear *Lhcb* promoter region. Among them, there were many response elements related to plant growth and development, including meristem expression (CAT-box element), light response element (Box4, CTT-motif, ATCT-motif, Ae-box, G-box, Kata-motif), circadian regulation, and endosperm expression (GCN4-motif). Furthermore, the promoter region contains hormone response elements, including those involved in auxin response (Aux RR-core element, TGA-element element), gibberellin response (P-box, GARE-motif), salicylic acid response (TCA-element), and methyl jasmonate response (TGACG-motif, CGTCA-motif). Additionally, stress response elements such as MYB binding sites, low-temperature response (LTR), defense and stress response elements (TC-rich repeats), and anaerobic induction (ARE) elements were also identified.

The promoter region of *PbrLhcb19* contained the highest number of abscisic acid response elements and also contained the most light response elements. In contrast, the promoter region of *PbrLhcb8* contained the most MeJA response elements. Most *PbrLhcb*s contain more than one abiotic stress response element, and the number of MYB-binding sites in the *PbrLhcb* promoter region is relatively high. It has been reported that MYB transcription factors respond to several stresses such as hormones, drought, high temperature, and high salt. The *Lhcb* gene in pear has more light-responsive elements, a feature common to all *Lhcb* genes. This feature suggests that light-sensitive reactions have a significant regulatory effect on *Lhcb* gene expression. Additionally, gibberellin, abscisic acid, salicylic acid, and auxin response elements were also identified. These results suggest that pear *Lhcb* genes are induced and regulated by stress and light regulation, which may be crucial for coping with stress.

### Distribution, expansion pattern, and collinearity analysis of *Lhcb* genes from nine Rosaceae species

*Lhcb* genes were located across all chromosomes in each of the investigated Rosaceae species, and the distribution of genes from chromosome to chromosome was uneven among the nine species (Fig. S3, S4). For example, in pear, *Lhcb* genes are primarily located on chromosomes 9 and 17, with lower distribution on other chromosomes. In strawberries, there are the most *Lhcb* genes (11) on chromosome 6. However, only one *Lhcb* gene was located on each chromosome 1, 2, and 4.

**Fig. 3:**
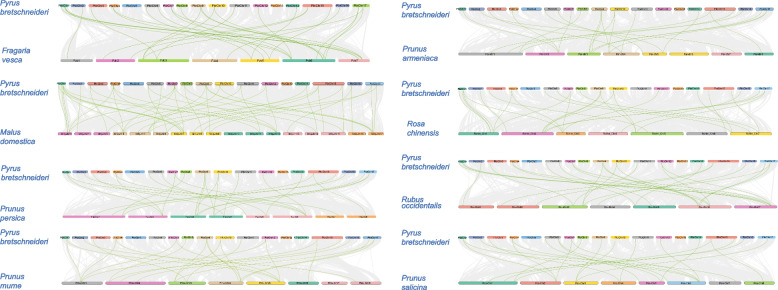
Collinearity analysis of *Lhcb* genes between eight rosaceae species and *Pyrus bretschneideri*. Syntenic relationships of *Lhcb* genes between *Pyrus bretschneideri* and *Fragaria vesca, Malus domestica, Prunus persica, Prunus mume, Prunus armeniaca, Rosa chinensis, Rubus occidentalis, Prunus salicina*

**Fig. 4:**
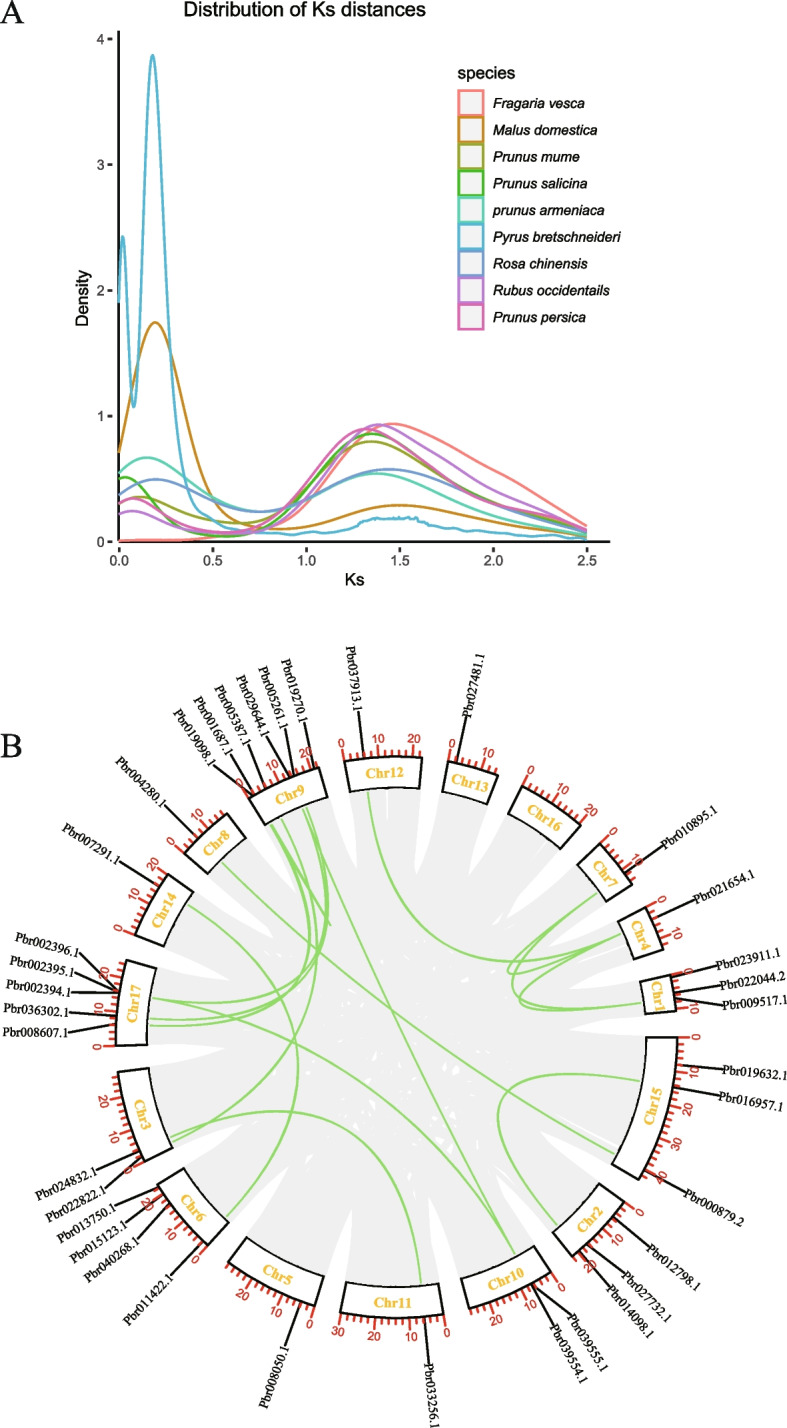
**A** Distribution of Ks distance in nine species of Rosaceae. **B** The collinearity of the *Lhc* gene in pear, and the green lines represent duplicate gene pairs

To further study duplication events throughout the evolutionary history of the *Lhcb* gene family using pear as a model, we analyzed the genome collinearity among nine Rosaceae species. Through analysis of the collinearity relationship between species, we determined that there is a high degree of collinearity between *Pyrus bretschneider* and *Malus domestica* but poor collinearity between *Pyrus bretschneider* and *Prunus armeniaca* (Fig. 3). These results demonstrated that pear and apple were closely related and strongly constrained by natural selection [37–41]. In contrast, pear and apricot may have undergone structural variations such as chromosome rearrangement, resulting in poor collinearity [39]. Visualization of collinearity between homologous *Lhcb* genes was performed to infer gene repetition events. Fifty-six duplicate gene pairs were identified in pear and apple, but only 28 homologous gene pairs could be matched between Pyrus bretschneider and other Rosaceae species, due to two shared WGD events occurring in apple and pear [42]. Additionally, the Ks values of the duplicate gene pairs varied between 0.10 and 1.94 (Table S3), indicating that duplicated gene pairs had evolved at different rates. The Ka/Ks comparison results showed that *PbrLhcbs* and *MdgLhcbs* were subjected to purifying selection. Purifying selection should theoretically eliminate harmful mutations in the population [43]. In woodland strawberry, the Ka to Ks ratio of FvLhcb11 was much higher than 1, suggesting that this gene was subject to strong positive selection and was rapidly evolving, which is of great significance for the evolution of the species. Many of the duplicated gene pairs experienced a WGD event, suggesting that WGD was critical in the expansion of *Lhcb* in Rosaceae (Fig. 4A). Simultaneously, we used the pear *Lhcb* gene family to analyze the intraspecies collinearity. The results indicated that the *Lhcb* gene was duplicated in series, and chromosome fragments were replicated. (Fig. 4B).

### *Lhcb* gene response to biotic and abiotic stresses

**Fig. 5:**
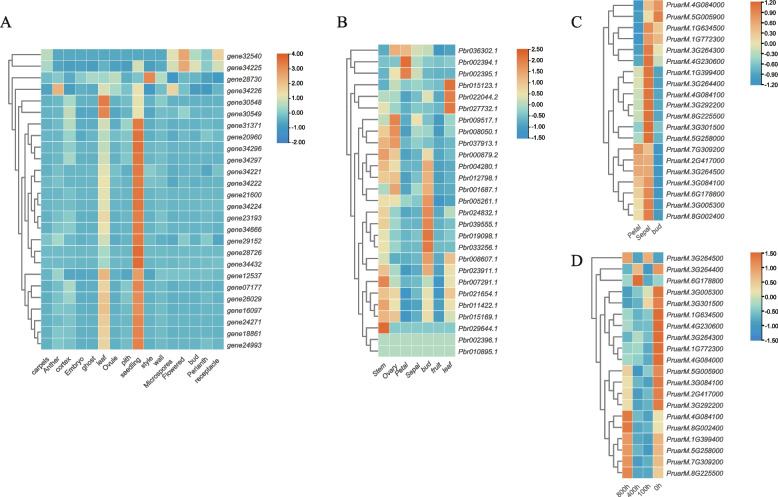
Expression profiles of *Lhcb* genes in different tissues and under different treatments. The bar at the right of each heatmap represents expression values. **A** Expression profiles of *FvLhcb* in 16 different tissues (bud, leaf, seedling, Anther, wall, cortex, pith, Microspores, carpels, perianth, flowered, receptacle, style, embryo, ghost, ovule). **B** Expression profiles of *PbrLhcb* in 7 different tissues (bud, stem, ovary, leaf, petal, sepal, fruit). **C** Expression profiles of *PruLhcb* in 3 different tissues(bud, sepal, petal). **D** The expression profiles of *PruLhcb* in buds treated at low temperature for different time points

**Fig. 6:**
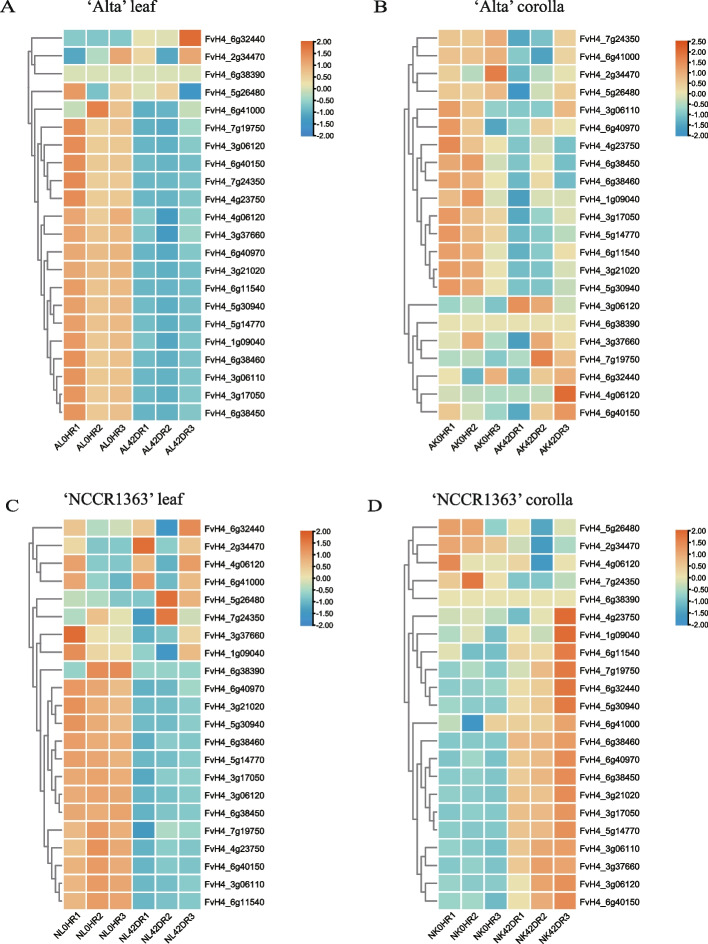
Heatmap of the expression profiles of *Lhcb* genes in strawberry in low temperature stresses. Leaves and corolla of two strawberry varieties were treated at low temperature. AL was the leaves of 'Alta' (**A**), AK was the corolla of 'Alta' (**B**), NL was the leaves of 'NCCR1363' (**C**), NK was the corolla of 'NCCR1363' (**D**). 0H represents the 0 h, 42D represents 42 days, R1-3 represents three repeats

We evaluated *Lhcb* gene expression patterns using several diverse transcriptome projects. We analyzed the *Lhcb* gene expression levels from 16 different strawberry tissues (bud, leaf, seedling, anther, wall, cortex, pith, microspores, carpels, perianth, flowered, receptacle, style, embryo, ghost, and ovule), seven different pear tissues (bud, stem, ovary, leaf, petal, sepal, and fruit), as well as three other apricot tissues (Fig. 5ABC). Results indicated that *Lhcb* exhibits tissue-specific expression. For example, in strawberries, many *Lhcb* genes were highly expressed in seedling bells, followed by the leaves. In pears, the overwhelming majority of *Lhcb* genes were highly expressed in shoots and stems, followed by leaves and ovaries. *Pbr002396.1* and *Pbr010895.1* were not expressed in any of the six tissues. In apricots, the *Lhcb* gene was expressed in all three tissues, and the expression level in the buds was relatively high. Low temperature may influence the chlorophyll content of plants, and we, therefore, studied the effect of low temperature on *PruLhcb* expression level in *Prunus armeniaca* buds (Fig. 5D). We determined that there were differences in *PruLhcb* expression levels between buds treated with the low temperature at different time points (Fig. 5D). Moreover, we investigated the effect of low temperature on the expression level of *FvLhcb* in the leaves and corolla of varying strawberry varieties ('NCCR1363' and 'Alta') (Fig. 6). The results demonstrated that *FvLhcb* genes responded differently to low temperatures, and there were differences across the different varieties. Under low-temperature stress, the expression of many *FvLhcb* genes in leaves decreased significantly. At the same time, gene *FvH4_2g34470* was up-regulated in both varieties, which may be due to the stress resistance of plants (Fig. 6A, C). However, *Lhcb* gene expression in NK (‘NCCR1363’ corolla) and AK (‘Alta’corolla) was distinct at low temperatures (Fig. 6B, D). This suggested that when the temperature decreased, the expression of chlorophyll a/b binding protein in leaves also decreased, influencing chlorophyll synthesis. It can also be concluded from these data that the genes are expressed in tissue-specific and species-specific manners.

### Real-time PCR analysis verifies the stress response of *Lhcb* genes

**Fig. 7:**
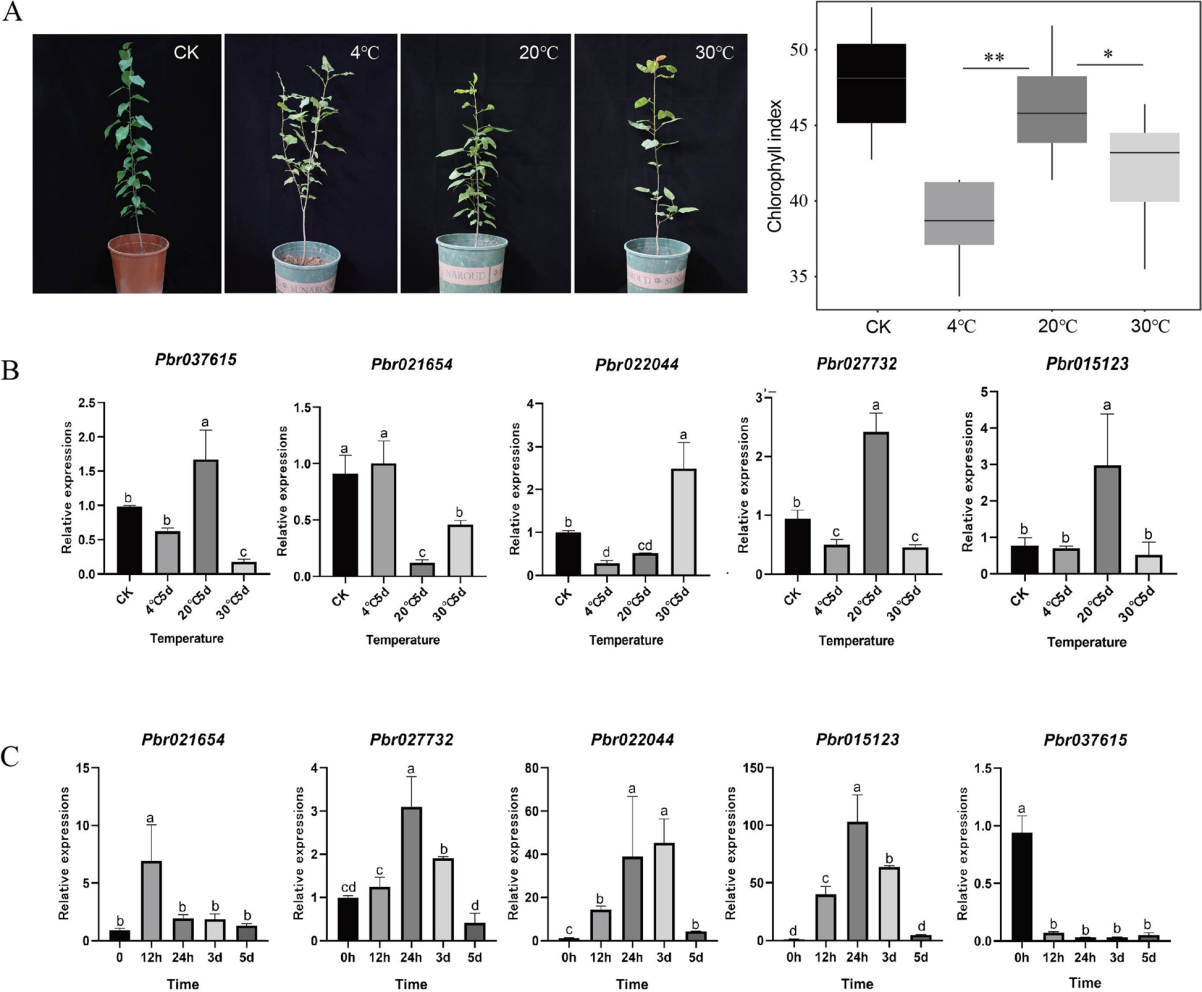
Phenotypic determination at different temperatures and qRT-PCR assay of key candidate genes identified in the heatmap. **A** The growth observation of pear seedlings and chlorophyll index determination of leaf cultured at three different temperatures of the fifth day. A double asterisk means extremely significant. **B** The relative expression of *PbrLhcb* at different temperatures. **C** Relative expression of *PbrLhcb* at 4℃ at the different time points. CK is an untreated sample growing in the greenhouse with the suitable environmental conditions. The experiments were repeated three times. The error bars represent mean ± SE (*n* = 3). The abcd in the figure represents the significance of the difference

'Douli' is an excellent rootstock for pear trees. In our study, 'Douli' pear seedlings with good growth and similar growth state were selected from the greenhouse to incubate in an artificial climate chamber at different temperatures (4℃, 20℃, 30℃). We observed that 4℃ and 30℃ treatments cause the chlorophyll content to decrease in pear leaves, and a higher chlorophyll content was presented in 20℃ treatment, indicating the most suitable growing temperature for pear seedling and low temperature significantly (*p*-value = 0.001) inhibit chlorophyll synthesis. Untreated pear seedling (CK) was growing in the greenhouse with the most suitable temperature, light, and ventilation condition, thus it presented a highest chlorophyll content (Fig. 7A). To further explore the response of *PbrLhcb* at different temperatures, genes with high expression levels were identified from the leaf tissues identified by RNA-seq data, and qPCR was performed on these selected genes. Under different temperature treatments, the relative expression levels of the three genes were the most elevated at 20℃. The relative expression levels of *Pbr021654* and *Pbr022044* were highest at 4℃ and 30℃, respectively (Fig. 7B). We then conducted cold treatment on plants to identify gene expression levels at different time periods. The experimental data indicated that the expression of four genes exhibited a similar trend of first increasing followed by a decrease after exposure to cold treatment (Fig. 7C). According to the results, the up-regulation of photosynthesis-related genes over the course of 0–24 h may be related to the influence of environmental conditions within a short period of time and the adaptation of plants to a low-temperature environment. Down-regulation over the course of 1 to 5 days may be due to increased sugar accumulation and superoxide dismutase and peroxidase activity due to longer cold treatments, which may inhibit the activity of these genes. Sugar-mediated inhibition of gene expression has been identified in genes associated with photosynthesis, including carbonic anhydrase [44], chlorophyll a/b binding protein [45], plastocyanin, and Rubisco small subunit [46]. Our experiment is consistent with these observed trends.

## Discussion

Light trapping chlorophyll a/b binding proteins (Lhc) are the most abundant protein complexes in thylakoid membranes, which play an important role in plant growth and development, including capture and transformation of light during photosynthesis and oxidative stress [15, 19, 20, 47, 48]. Lhc superfamily contains a variety of chlorophyll and carotenoids binding protein, playing an important role in capturing light and protecting green plants and algae [2, 49–51]. In green plants, these proteins point to chloroplasts or plastids. Although the similarity of the overall sequence may be low, but the characteristic of proteins in this superfamily is the chlorophyll binding domain located in the thylakoid membrane [3, 19, 51]. So far, four related higher plant subfamilies of the Lhc superfamily have been described including Lhc, PsbS, Lil, and FCII. Furthermore, a new family, RedCAP (red lineage chlorophyll a /b-binding-like), has been discovered in *Rhodophyta* and *Bacillariophyta *[51]. The Lil family is more primitive [50, 51]. OHP1 (Lil1) is one of the subfamilies, and cyanobacteria is one of the eukaryotes with a long evolutionary history. The presence of sequences similar to OHP1 in blue-green algae indicates that the single helix protein is primitive. After primary endophytic HLIP The encoded plastids tend to transfer to the nuclear genome and are lost in the common ancestor of all botanical families [50, 51]. In the course of later evolution, it is possible that members of the light-harvesting family expanded through gene replication in order to adapt to the environment for better photosynthesis. This may have led to an expansion of the Lhc family. Lhcb is one of the members of the Lhc superfamily and has been identified and characterized in many plants. Several studies have demonstrated that each protein complex has specific functions under natural environmental conditions [2, 3, 8]. In Arabidopsis, thermal energy dissipation is a central photo-protection mechanism in response to environmental stresses [52–55]. The antenna system is involved in the light-energy dissipation [47, 56, 57]; previous studies on Lhcb4 and Lhcb6 have demonstrated that they may take part in nonphotochemical dissipation of superfluous energy [56, 58–60]. It has been established that Lhcb5 may be the key factor in the catalysis of qI quenching [14, 61–64]. It has been demonstrated that Lhcb6 deficiency may lead to severe oxidative damage [64]. Loss of Lhcb1 has been reported to induce a compensatory mechanism in plants, including kinases and phosphatases, regulating photosynthetic ETC balance [65, 66]. The primary function of Lhcb3 is to modulate state transitions. Phosphorylation of the Light-Harvesting Complex II isoform Lhcb2 is crucial for state transitions. Compared to Lhcb1, the higher phosphorylation level and similar phosphorylation dynamics indicate that Lhcb2 is preferentially phosphorylated and is a better substrate for kinases in terms of accessibility or recognition. Lhcb protein has been extensively studied in *Arabidopsis thaliana*. Therefore, we aimed to infer the function of the Lhcb protein in the Rosaceae family based on the reported function of Lhcb in *Arabidopsis thaliana*. Gene duplication is a universal phenomenon taking place in plant evolution that allows for the accumulation of new functions [6, 9, 20, 28, 67, 68]. These duplicates frequently occur in segmental, whole-genome, and tandem duplication events [69]. Within the *Lhcb* gene family, *Lhcb7* is common in higher plants, encoding transcripts that are highly expressed in a subpopulation of mesophyllal cells and associated with protein products homologous to pigment binding components in the photosystem (PSII) peripheral antenna complex [14, 15, 63, 69, 70]. In our study, we identified 33, 29, 21, 15, 20, 20, 33, 23 and 18 Lhcb genes in apple, pear, peach, Japanese Plum, apricot, Japanese apricot, Chinese rose, strawberry and black raspberry, respectively. In nine Rosaceae species, lineage-specific replication was more effective in than species-specific replication in *Lhcb* gene amplification. The study found that recent duplication of genome-wide replication events produced similar Ks peaks (0.1 to 0.2) in the Lhcb gene family of apple and pear. Additionally, a small number of Lhcb genes underwent tandem duplication and were located in across all chromosomes of nine Rosaceae species. Furthermore, Lhcb genes with Ka/Ks less than 1 indicate that they may be developing to new functions and being driven by selective pressure. In our study, phylogenetic tree demonstrated that the Lhcb family is divided into seven proteins (Lhcb1, Lhcb2, Lhcb3, Lhcb4, Lhcb5, Lhcb6, and Lhcb7). The Lhcb protein is conserved in a single clade and may have similar functions. The results of expression analysis showed that *Lhcb* gene was specifically expressed in different tissues after exposure to different stress. However, the differential expression patterns were mainly concentrated among different subfamily genes. As the number of gene family members in a species increased, the differentiation of expression patterns was more obvious [71]. The expression of the Lhcb gene family in strawberry was the highest at the seedling stage, followed by the leaf, and the lowest in the embryo. The expression of the Lhcb gene family was also varied in different tissues of the pear, with high expression in the bud and stem, followed by leaves, and no expression in the fruit. This distribution is consistent with the idea that Lhcb is expressed in the green portions of plants as a light-trapping pigment-binding protein gene, and chloroplasts are the light response sites in photosynthesis. Photosynthesis occurs predominantly in the leaves, buds, and stems of plants. Pbr029644.1 was highly expressed in the young stem of pears but sparingly expressed in the rest of the plant, suggesting that this gene may be required for photosynthesis in the stem. Analysis of Lhcb family members responding to low-temperature stress demonstrated that the expression level of FvLhcb in leaves decreased under stress. In contrast, the expression level of FVLhcb in the corolla increased. Therefore, we hypothesized that when plants were stressed, they would preferentially progress in their life cycle. By qPCR, we determined that the expression of PbrLhcbs were the highest at 20℃, which was a relatively suitable temperature for plant growth. However, the lack of multiple median temperatures limited our determination of the optimal temperature for plant growth. Additionally, we observed relatively lower chlorophyll content of pear leaves in 4℃ and 30℃ treatmants, and a highest chlorophyll content was observed in 20℃ treatment, indicating low or high temperature enables the synthesis inhibition and rapid degradation of chlorophyll. According to qPCR analysis results at different time periods, PbrLhcbs exhibited a trend of initially increasing and then decreasing. This may be due to the sudden changes in temperature and the requirement for plants to adapt to new environments. Over a short period of time, plants require a lot of energy, and PbrLhcbs initially increases through chlorophyll synthesis, driving photosynthesis to provide the nutrients necessary for life. After adaptation to the new environment, gene expression was reduced but still higher than the previous untreated gene expression. This indicates that these genes are involved in stress response.

## Conclusion

We identified *Lhcb* genes from nine Rosaceae species, and analyzed their phylogenetic tree, family expansion, cis-acting elements, and expression patterns of different tissues and environmental stress, aiming to increase our understanding of the mechanisms underlying the evolution and responses to stress of *Lhcb* gene family in Rosaceae fruit crops. Sequences analysis showed that *Lhcb* genes are highly conservative in the Lhcb domain between species in Rosaceae. Through evolutionary analysis, it has been observed that the Lhcb protein is highly conserved within the Lhcb3 branch, suggesting potential functional similarities among its members. Analysis of the upstream cis-element results in the *PbrLhcbs* gene has revealed associations with stress response, hormone response, and light response. This indicates that the *Lhcb* gene family is indispensable in various aspects of plant growth and development. The expression patterns of the *Lhcb* gene family in the leaves of strawberry, pear, and Japanese Plum underscores its crucial roles in plant growth and development, and *Lhcb* genes were predominantly present in various plant tissues such as leaves, flower buds, leaf buds, tender stems, and seedlings, indicating their importance in plant morphogenesis. Under low-temperature treatment, the synthesis of chlorophyll was suppressed and the expression of *Lhcb* genes initially increased and then decreased, indicating a potential involvement in stress response. These findings contribute to a better understanding of the biological function of the *Lhcb* gene family and will pave the way for further resistance breeding of fruit crops.

### Supplementary Information

**Additional file 1: Fig. S1.** Multiple sequence alignment of the LHCB domain. Multiple sequence analysis of LHCB gene in pear and peach (A). Sequence markers of repeated sequences are based on full-length alignment of all Arabidopsis LHCB domains. Multiple comparison analysis of 290 LHCB domains was performed using ClustalW. The bit fraction indicates the content of the information at each position in the sequence (B).

**Additional file 2: Fig. S2.** Protein domain of the LHCB. Different color regions represent different species, and these domains all belong to the chlorophyll a/b binding protein. PLN00147, PLN00025, PLN00101 PLN00170, PLN00187,PLN00048,PLN00101 belong to cl02879 superfamiliy.PLN00171 belongs to the cl29582 superfamily.

**Additional file 3: Fig. S3.** Localization of LHCB gene in Rosaceae chromosomes. Different colours represent different species, green is Rubus occidentalis (A); Blue is Rosa chinensis (B); In brown is Prunus armeniaca (C); In pink is Prunus.mume (D); Yellow is Pyrus bretschneider (E).

**Additional file 4: Fig. S4.** Localization of LHCB gene in Rosaceae chromosomes. Different colours represent different species, purple is Prunus.salicina (A); In blue, Fragaria vesca (B); Green is Prunus persica (C); The gray is Malus domestica (D).

**Additional file 5: Table S1-S6.**

## Data Availability

Raw sequence data of Prunus armeniaca and Fragaria Vesca was downloaded from the NCBI database using accession number PRJNA577143 and PRJNA700642 (https://www.ncbi.nlm.nih.gov/geo/). All datasets generated in this study are included in the published article/Additional Files. Websites used for analyses in this study are as follows: TAIR (https://www.arabidopsis.org/), GDR (https://www.rosaceae.org/), HMMER 3.0 (http://hmmer.janelia.org/), PfamScan and Pfam A (http://pfam.xfam.org/), ExPASy (http://web.expasy.org/protparam/), R (https://cran.r-project.org), STRING (http://string-db.org/), KaKs Calculator (http://code.google.com/p/kaks-calculator/wiki/kaks_Calculator). Access to these databases or websites is open. No new sequence data was generated in this study.
