# Correction: Comparative genomics analysis provides insights into evolution and stress responses of *Lhcb* genes in Rosaceae fruit crops

**DOI:** 10.1186/s12870-023-04667-0

**Published:** 2023-12-14

**Authors:** Xiaolong Li, Zeyu Jiang, Chaofan Zhang, Kefan Cai, Hui Wang, Weiyi Pan, Xuepeng Sun, Yongbin Gao, Kai Xu

**Affiliations:** 1 Key Laboratory of Quality and Safety Control for Subtropical Fruit and Vegetable, Ministry of Agriculture and Rural Affairs, College of Horticulture Science, Zhejiang A&F University, Hangzhou, 311300 Zhejiang China; 2 College of Agriculture and Biotechnology, Zhejiang University, Hangzhou, 310058 Zhejiang China

**Correction:** ***J Exp Clin Cancer Res*** **23, 484 (2023)**

10.1186/s12870-023-04438-x

Following publication of the original article [1], the authors would like to correct the Background of the Abstract section.

Background should be:

Light-harvesting chlorophyll a/b binding proteins (Lhcb) play crucial roles in plant growth, development, and the response to abiotic stress in higher plants. Previous studies have reported that *Lhcb* genes were involved in the phytochrome regulation and responded to different light and temperature conditions in Poaceae (such as maize). However, the evolution and functions of *Lhcb* genes remains poorly characterized in important Rosaceae species.

The correction does not affect the overall result or conclusion of the article. The original article has been corrected.
