# Supplementary material for: Comparative genomics analysis provides insights into evolution and stress responses of Lhcb genes in Rosaceae fruit crops
Source: BMC Plant Biol. 2023 Oct 11;23:484. doi: 10.1186/s12870-023-04438-x (PMC10566169; doi:10.1186/s12870-023-04438-x)
Supplement: Supplementary file 1 — Additional file 1: Fig. S1. Multiple sequence alignment of the LHCB domain. Multiple sequence analysis of LHCB gene in pear and peach (A). Sequence markers of repeated sequences are based on full-length alignment of all Arabidopsis LHCB domains. Multiple comparison analysis of 290 LHCB domains was performed using ClustalW. The bit fraction indicates the content of the information at each position in the sequence (B). [file 12870_2023_4438_MOESM1_ESM.pdf]

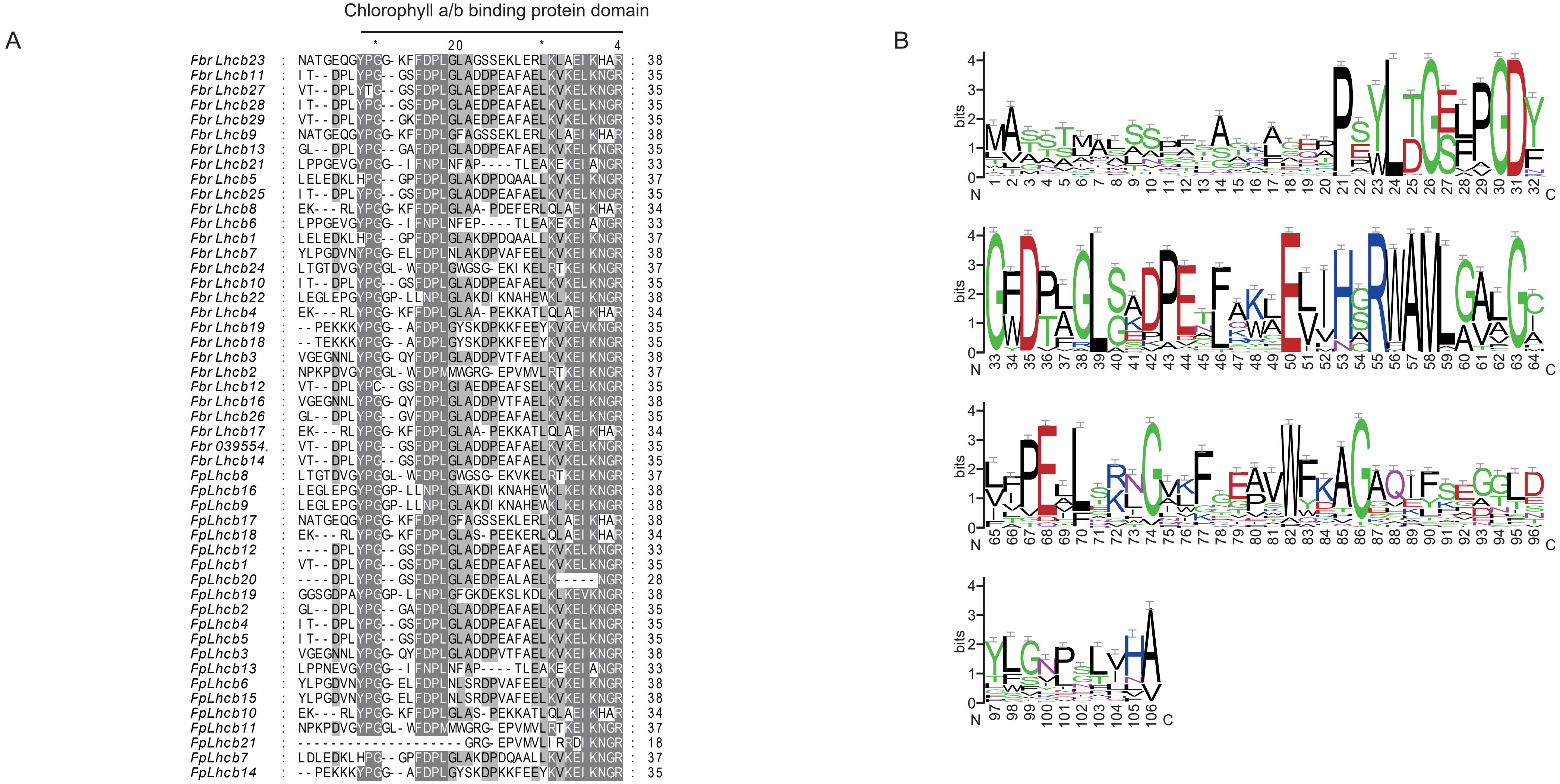

Fig.S1Multiple sequence alignment of the LHCB domain. Multiple sequence analysis of LHCB gene in pear and peach (A).Sequence markers of repeated sequences are based on full-length alignment of all Arabidopsis LHCB domains. Multiple comparison analysis of 290 LHCB domains was performed using ClustalW. The bit fraction indicates the content of the information at each position in the sequence(B).
