# Supplementary material for: Comparative genomics analysis provides insights into evolution and stress responses of Lhcb genes in Rosaceae fruit crops
Source: BMC Plant Biol. 2023 Oct 11;23:484. doi: 10.1186/s12870-023-04438-x (PMC10566169; doi:10.1186/s12870-023-04438-x)
Supplement: Supplementary file 2 — Additional file 2: Fig. S2. Protein domain of the LHCB. Different color regions represent different species, and these domains all belong to the chlorophyll a/b binding protein. PLN00147, PLN00025, PLN00101 PLN00170, PLN00187,PLN00048,PLN00101 belong to cl02879 superfamiliy.PLN00171 belongs to the cl29582 superfamily. [file 12870_2023_4438_MOESM2_ESM.pdf]

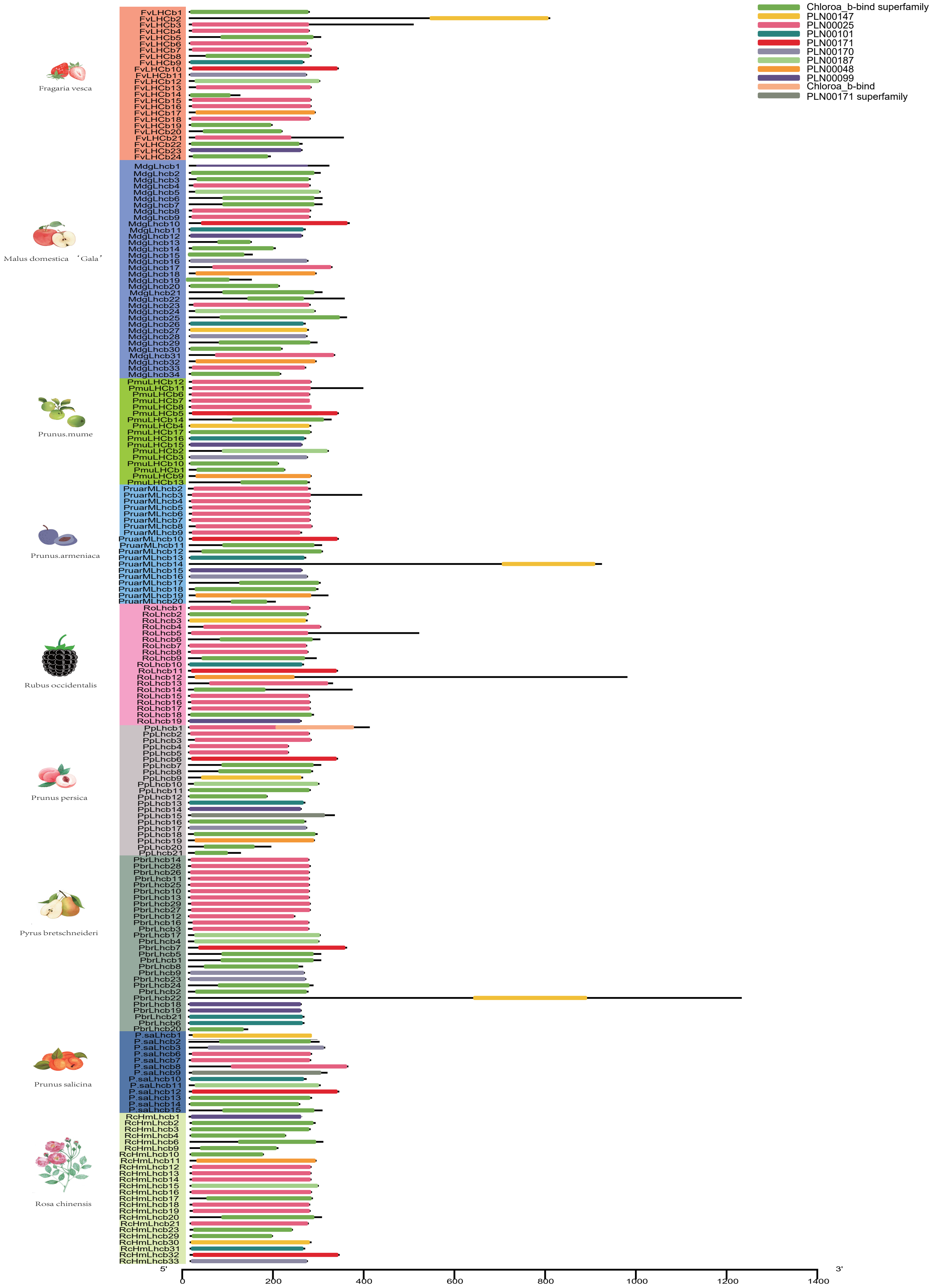

Fig S2. Protein domain of the LHCB. Different color regions represent different species, and these domains all belong to the chlorophyll a/b binding protein. PLN00147, PLN00025, PLN00101 PLN00170, PLN00187,PLN00048,PLN00101 belong to cl02879 superfamiliy.PLN00171 belongs to the cl29582 superfamily.
