# Supplementary material for: Comparative genomics analysis provides insights into evolution and stress responses of Lhcb genes in Rosaceae fruit crops
Source: BMC Plant Biol. 2023 Oct 11;23:484. doi: 10.1186/s12870-023-04438-x (PMC10566169; doi:10.1186/s12870-023-04438-x)
Supplement: Supplementary file 3 — Additional file 3: Fig. S3. Localization of LHCB gene in Rosaceae chromosomes. Different colours represent different species, green is Rubus occidentalis (A); Blue is Rosa chinensis (B); In brown is Prunus armeniaca (C); In pink is Prunus.mume (D); Yellow is Pyrus bretschneider (E). [file 12870_2023_4438_MOESM3_ESM.pdf]

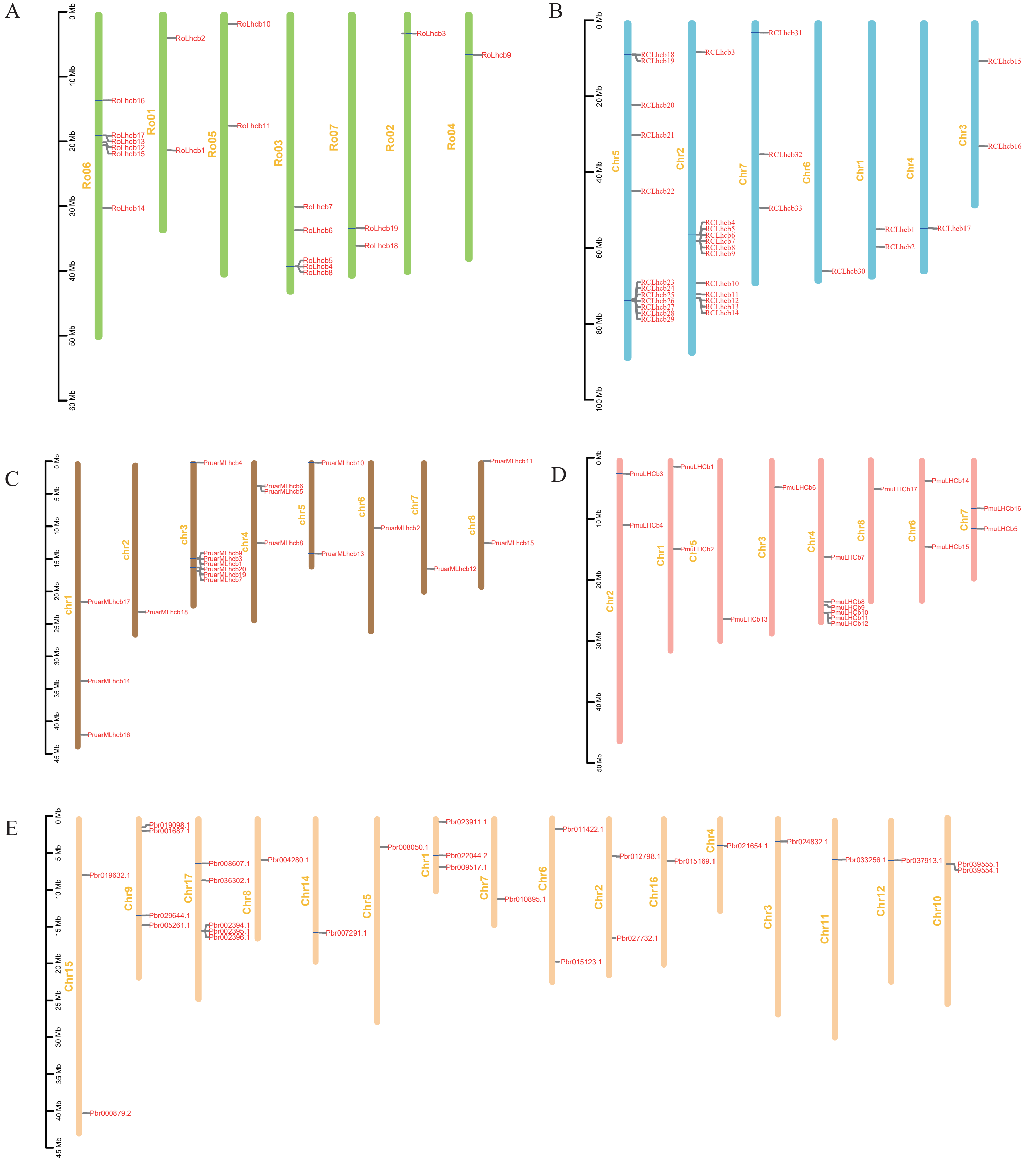

Fig.S3 Localization of LHCB gene in Rosaceae chromosomes. Different colours represent different species, green is *Rubus occidentalis* (A); Blue is *Rosa chinensis* (B); In brown is *Prunus armeniaca* (C); In pink is *Prunus.mume* (D); Yellow is *Pyrus bretschneider* (E).
