# Supplementary material for: Comparative genomics analysis provides insights into evolution and stress responses of Lhcb genes in Rosaceae fruit crops
Source: BMC Plant Biol. 2023 Oct 11;23:484. doi: 10.1186/s12870-023-04438-x (PMC10566169; doi:10.1186/s12870-023-04438-x)
Supplement: Supplementary file 4 — Additional file 4: Fig. S4. Localization of LHCB gene in Rosaceae chromosomes. Different colours represent different species, purple is Prunus.salicina (A); In blue, Fragaria vesca (B); Green is Prunus persica (C); The gray is Malus domestica (D). [file 12870_2023_4438_MOESM4_ESM.pdf]

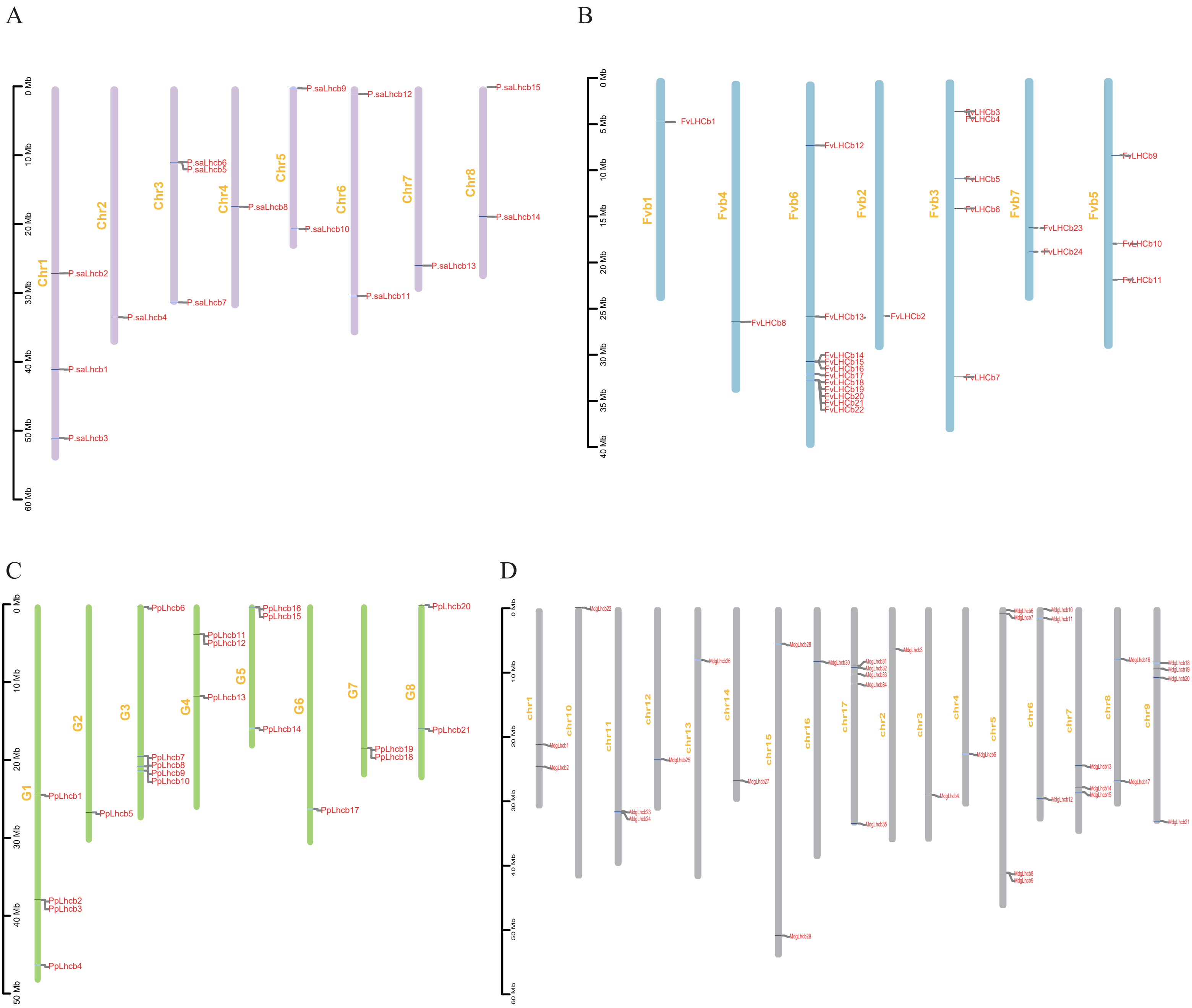

Fig.S4 Localization of LHCB gene in Rosaceae chromosomes. Different colours represent different species, purple is Prunus.salicina (A); In blue, Fragaria vesca (B); Green is Prunus persica (C); The gray is Malus domestica (D).
