# Supplementary material for: Comparative genomics analysis provides insights into evolution and stress responses of Lhcb genes in Rosaceae fruit crops
Source: BMC Plant Biol. 2023 Oct 11;23:484. doi: 10.1186/s12870-023-04438-x (PMC10566169; doi:10.1186/s12870-023-04438-x)
Supplement: Supplementary file 5 — Additional file 5: Table S1-S6. [file 12870_2023_4438_MOESM5_ESM.zip › Supplemental Tables/Table S1.docx]

| **Table S1 results of Lhcb hmmserach** | | | | | | | | | |
| --- | --- | --- | --- | --- | --- | --- | --- | --- | --- |
| E-value | score | bias | E-value | score | bias | exp | N | Sequence | Renamed |
| 1.3E-15 | 58.7 | 10.7 | 1.7E-08 | 35.3 | 0.4 | 3.2 | 5 | AT1G15820.1 | AtLHCb1 |
| 9.5E-10 | 39.4 | 1.8 | 0.00033 | 21.3 | 0.1 | 3 | 3 | AT1G29910.1 | AtLHCb2 |
| 9.5E-10 | 39.4 | 1.8 | 0.00033 | 21.3 | 0.1 | 3 | 3 | AT1G29920.1 | AtLHCb3 |
| 1.9E-09 | 38.5 | 2.2 | 0.00034 | 21.3 | 0.1 | 3 | 3 | AT1G29930.1 | AtLHCb4 |
| 3.2E-10 | 41 | 0.5 | 0.0004 | 21 | 0.1 | 3 | 3 | AT1G76570.1 | AtLHCb5 |
| 6.5E-07 | 30.2 | 1 | 0.001 | 19.7 | 0.1 | 2.9 | 3 | AT2G05070.1 | AtLHCb6 |
| 1.7E-09 | 38.6 | 2.6 | 0.00035 | 21.2 | 0.1 | 3 | 3 | AT2G34420.1 | AtLHCb7 |
| 3.4E-09 | 37.6 | 2 | 0.00031 | 21.4 | 0.1 | 3 | 3 | AT2G34430.1 | AtLHCb8 |
| 8.2E-125 | 415.8 | 0 | 1E-123 | 412.2 | 0 | 1.9 | 1 | AT2G40100.1 | AtLHCb9 |
| 3.3E-106 | 354.9 | 0.1 | 3.9E-106 | 354.7 | 0.1 | 1 | 1 | AT2G40100.2 | AtLHCb10 |
| 1.2E-139 | 464.3 | 0 | 1.4E-139 | 464.1 | 0 | 1 | 1 | AT3G08940.1 | AtLHCb11 |
| 2.6E-123 | 410.9 | 0.1 | 9.4E-122 | 405.8 | 0.1 | 2 | 1 | AT3G08940.2 | AtLHCb12 |
| 9.9E-07 | 29.6 | 0.9 | 0.0015 | 19.1 | 0.1 | 2.9 | 3 | AT3G27690.1 | AtLHCb13 |
| 0.0000016 | 28.9 | 1 | 0.002 | 18.7 | 0.1 | 2.9 | 3 | AT3G27690.2 | AtLHCb14 |
| 9.9E-13 | 49.2 | 7.1 | 3.3E-08 | 34.4 | 1.4 | 3.7 | 3 | AT4G10340.1 | AtLHCb15 |
| 1.8E-112 | 375.4 | 0.1 | 7.1E-111 | 370.2 | 0.1 | 2 | 1 | AT5G01530.1 | AtLHCb16 |
| 0.0000001 | 32.8 | 1 | 0.00053 | 20.6 | 0.1 | 3.2 | 3 | AT5G54270.1 | AtLHCb17 |
| 9.1E-27 | 95.2 | 0 | 9.8E-27 | 95 | 0 | 1 | 1 | Pbr000879.2 | PbrLHCb1 |
| 6.7E-50 | 171.4 | 0 | 7.7E-50 | 171.2 | 0 | 1 | 1 | Pbr001687.1 | PbrLHCb2 |
| 1.9E-48 | 166.5 | 0 | 2.1E-48 | 166.4 | 0 | 1 | 1 | Pbr002394.1 | PbrLHCb3 |
| 2.5E-50 | 172.7 | 0 | 2.8E-50 | 172.6 | 0 | 1 | 1 | Pbr002395.1 | PbrLHCb4 |
| 1.8E-48 | 166.7 | 0 | 2E-48 | 166.5 | 0 | 1 | 1 | Pbr002396.1 | PbrLHCb5 |
| 9.7E-28 | 98.4 | 0 | 1.1E-27 | 98.2 | 0 | 1 | 1 | Pbr004280.1 | PbrLHCb6 |
| 2.1E-47 | 163.2 | 0 | 2.4E-47 | 162.9 | 0 | 1 | 1 | Pbr005261.1 | PbrLHCb7 |
| 1E-12 | 48.9 | 0 | 1.6E-12 | 48.2 | 0 | 1.3 | 1 | Pbr007291.1 | PbrLHCb8 |
| 4.2E-14 | 53.4 | 0 | 1.8E-13 | 51.3 | 0 | 1.7 | 1 | Pbr008050.1 | PbrLHCb9 |
| 6.7E-50 | 171.4 | 0 | 7.7E-50 | 171.2 | 0 | 1 | 1 | Pbr008607.1 | PbrLHCb10 |
| 5.6E-14 | 53 | 0 | 8.5E-14 | 52.4 | 0 | 1.2 | 1 | Pbr011422.1 | PbrLHCb11 |
| 6.5E-15 | 56.1 | 0 | 5.1E-14 | 53.2 | 0 | 1.9 | 1 | Pbr012798.1 | PbrLHCb12 |
| 6.9E-07 | 29.7 | 0 | 0.000001 | 29.1 | 0 | 1.2 | 1 | Pbr013750.1 | PbrLHCb13 |
| 4.3E-09 | 36.9 | 0 | 6.1E-09 | 36.4 | 0 | 1.1 | 1 | Pbr014098.1 | PbrLHCb14 |
| 2E-12 | 47.9 | 0 | 1.8E-11 | 44.8 | 0 | 2 | 1 | Pbr015123.1 | PbrLHCb15 |
| 6.1E-38 | 132 | 0 | 7.5E-38 | 131.7 | 0 | 1.1 | 1 | Pbr015169.1 | PbrLHCb16 |
| 2.7E-10 | 40.9 | 0.1 | 3.9E-10 | 40.4 | 0.1 | 1.1 | 1 | Pbr016957.1 | PbrLHCb17 |
| 6.7E-50 | 171.4 | 0 | 7.7E-50 | 171.2 | 0 | 1 | 1 | Pbr019098.1 | PbrLHCb18 |
| 1.9E-22 | 80.9 | 0 | 9.1E-22 | 78.7 | 0 | 1.8 | 2 | Pbr019270.1 | PbrLHCb19 |
| 7.1E-07 | 29.6 | 0 | 0.0015 | 18.7 | 0 | 2.3 | 2 | Pbr019632.1 | PbrLHCb20 |
| 1.7E-10 | 41.5 | 0 | 2.3E-10 | 41.1 | 0 | 1.1 | 1 | Pbr022822.1 | PbrLHCb21 |
| 4.8E-33 | 115.8 | 0 | 5.6E-33 | 115.6 | 0 | 1 | 1 | Pbr024832.1 | PbrLHCb22 |
| 1.1E-12 | 48.8 | 0 | 1.5E-12 | 48.3 | 0 | 1.2 | 1 | Pbr027481.1 | PbrLHCb23 |
| 3.7E-20 | 73.4 | 0 | 4.5E-20 | 73.1 | 0 | 1.1 | 1 | Pbr027732.1 | PbrLHCb24 |
| 3.3E-45 | 155.9 | 0 | 3.6E-45 | 155.8 | 0 | 1 | 1 | Pbr029644.1 | PbrLHCb25 |
| 1.3E-33 | 117.7 | 0 | 1.6E-33 | 117.4 | 0 | 1 | 1 | Pbr033256.1 | PbrLHCb26 |
| 4.7E-48 | 165.3 | 0 | 5.7E-48 | 165 | 0 | 1 | 1 | Pbr036302.1 | PbrLHCb27 |
| 8.6E-47 | 161.1 | 0 | 1.7E-40 | 140.4 | 0 | 2 | 2 | Pbr039554.1 | PbrLHCb28 |
| 2.6E-47 | 162.8 | 0 | 3.1E-47 | 162.6 | 0 | 1 | 1 | Pbr039555.1 | PbrLHCb29 |
| 3.50E-111 | 371.4 | 0.1 | 4.00E-111 | 371.2 | 0.1 | 1 | 1 | FvH4_1g09040.1 | FvLHCb1 |
| 2.10E-110 | 368.9 | 0.1 | 2.40E-110 | 368.7 | 0.1 | 1 | 1 | FvH4_2g34470.1 | FvLHCb2 |
| 4.10E-110 | 367.9 | 0.1 | 4.70E-110 | 367.7 | 0.1 | 1 | 1 | FvH4_3g06110.1 | FvLHCb3 |
| 2.70E-109 | 365.2 | 0.1 | 3.30E-109 | 364.9 | 0.1 | 1 | 1 | FvH4_3g06120.1 | FvLHCb4 |
| 5.60E-108 | 360.9 | 0.1 | 6.70E-108 | 360.6 | 0.1 | 1 | 1 | FvH4_3g17050.1 | FvLHCb5 |
| 8.00E-108 | 360.4 | 0.1 | 1.10E-107 | 359.9 | 0.1 | 1.1 | 1 | FvH4_3g21020.1 | FvLHCb6 |
| 1.90E-106 | 355.9 | 0 | 2.20E-106 | 355.7 | 0 | 1 | 1 | FvH4_3g37660.1 | FvLHCb7 |
| 1.10E-99 | 333.7 | 0.2 | 1.20E-99 | 333.5 | 0.2 | 1 | 1 | FvH4_4g23750.1 | FvLHCb8 |
| 2.30E-99 | 332.6 | 0.2 | 3.20E-99 | 332.2 | 0.2 | 1.1 | 1 | FvH4_5g14770.1 | FvLHCb9 |
| 3.30E-91 | 306 | 0 | 3.80E-91 | 305.7 | 0 | 1 | 1 | FvH4_5g26480.1 | FvLHCb10 |
| 1.60E-89 | 300.4 | 0 | 1.80E-89 | 300.2 | 0 | 1 | 1 | FvH4_5g30940.1 | FvLHCb11 |
| 1.00E-84 | 284.7 | 0 | 1.30E-84 | 284.4 | 0 | 1.1 | 1 | FvH4_6g11540.1 | FvLHCb12 |
| 2.70E-83 | 280 | 0.1 | 4.20E-83 | 279.4 | 0.1 | 1.3 | 1 | FvH4_6g32440.1 | FvLHCb13 |
| 5.20E-82 | 275.8 | 0.1 | 6.10E-82 | 275.6 | 0.1 | 1 | 1 | FvH4_6g38390.1 | FvLHCb14 |
| 8.30E-82 | 275.1 | 0.3 | 9.70E-82 | 274.9 | 0.3 | 1 | 1 | FvH4_6g38450.1 | FvLHCb15 |
| 2.10E-80 | 270.5 | 0.1 | 2.50E-80 | 270.3 | 0.1 | 1.1 | 1 | FvH4_6g38460.1 | FvLHCb16 |
| 4.00E-79 | 266.3 | 0.1 | 5.10E-79 | 266 | 0.1 | 1 | 1 | FvH4_6g40150.1 | FvLHCb17 |
| 8.30E-75 | 252.2 | 1.2 | 1.00E-74 | 251.9 | 1.2 | 1 | 1 | FvH4_6g40970.1 | FvLHCb18 |
| 1.60E-65 | 221.8 | 0.3 | 8.40E-61 | 206.3 | 0 | 2 | 2 | FvH4_6g41000.1 | FvLHCb19 |
| 1.90E-62 | 211.7 | 0.2 | 3.10E-38 | 132.4 | 0 | 2.1 | 2 | FvH4_6g41020.1 | FvLHCb20 |
| 5.50E-61 | 206.9 | 0.1 | 6.60E-61 | 206.7 | 0.1 | 1.1 | 1 | FvH4_6g41050.1 | FvLHCb21 |
| 2.60E-49 | 168.7 | 0 | 3.20E-49 | 168.3 | 0 | 1 | 1 | FvH4_6g41060.1 | FvLHCb22 |
| 5.20E-37 | 128.3 | 0 | 5.80E-37 | 128.2 | 0 | 1 | 1 | FvH4_7g19750.1 | FvLHCb23 |
| 5.90E-29 | 102 | 0.1 | 7.40E-29 | 101.6 | 0.1 | 1.1 | 1 | FvH4_7g24350.1 | FvLHCb24 |
| 2.50E-111 | 371.9 | 0.1 | 2.90E-111 | 371.7 | 0.1 | 1 | 1 | PmuVarChr43241 | PmuLHCb12 |
| 1.40E-110 | 369.4 | 0.1 | 1.80E-110 | 369.1 | 0.1 | 1.1 | 1 | PmuVarChr43240 | PmuLHCb11 |
| 7.40E-109 | 363.8 | 0.1 | 8.90E-109 | 363.6 | 0.1 | 1 | 1 | PmuVarChr30795 | PmuLHCb6 |
| 7.50E-109 | 363.8 | 0.1 | 9.00E-109 | 363.5 | 0.1 | 1 | 1 | PmuVarChr30796 | PmuLHCb7 |
| 5.80E-106 | 354.3 | 0.1 | 6.80E-106 | 354.1 | 0.1 | 1 | 1 | PmuVarChr43015 | PmuLHCb8 |
| 1.30E-102 | 343.3 | 0.5 | 5.20E-102 | 341.4 | 0.5 | 1.7 | 1 | PmuVarChr41827 | PmuLHCb7 |
| 2.10E-100 | 336.1 | 0.5 | 2.50E-100 | 335.9 | 0.5 | 1 | 1 | PmuVarChr32057 | PmuLHCb8 |
| 1.40E-91 | 307.2 | 0 | 1.70E-91 | 307 | 0 | 1 | 1 | PmuVarChr71684 | PmuLHCb5 |
| 2.00E-90 | 303.4 | 0 | 2.40E-90 | 303.2 | 0 | 1 | 1 | PmuVarChr60368 | PmuLHCb14 |
| 2.70E-85 | 286.6 | 0.1 | 3.30E-85 | 286.3 | 0.1 | 1.1 | 1 | PmuVarChr23416 | PmuLHCb5 |
| 3.40E-84 | 283 | 0.1 | 4.10E-84 | 282.8 | 0.1 | 1 | 1 | PmuVarChr21407 | PmuLHCb4 |
| 5.40E-84 | 282.4 | 0.2 | 6.30E-84 | 282.1 | 0.2 | 1 | 1 | PmuVarChr80915 | PmuLHCb17 |
| 3.60E-82 | 276.4 | 0.1 | 4.00E-82 | 276.2 | 0.1 | 1 | 1 | PmuVarChr71167 | PmuLHCb16 |
| 1.20E-79 | 268.1 | 0.1 | 1.50E-79 | 267.8 | 0.1 | 1 | 1 | PmuVarChr61963 | PmuLHCb15 |
| 5.00E-79 | 266.1 | 0 | 6.10E-79 | 265.8 | 0 | 1.1 | 1 | PmuVarChr11667 | PmuLHCb2 |
| 4.40E-76 | 256.4 | 1.3 | 5.40E-76 | 256.1 | 1.3 | 1 | 1 | PmuVarChr20297 | PmuLHCb3 |
| 7.30E-68 | 229.5 | 0.6 | 1.20E-39 | 137 | 0 | 2.1 | 2 | PmuVarChr43239 | PmuLHCb10 |
| 2.40E-61 | 208.2 | 0.7 | 1.10E-48 | 166.6 | 0 | 2.3 | 2 | PmuVarChr10116 | PmuLHCb1 |
| 6.80E-58 | 196.8 | 0.1 | 7.90E-58 | 196.6 | 0.1 | 1 | 1 | PmuVarChr43096 | PmuLHCb9 |
| 1.40E-54 | 186 | 0.1 | 2.00E-54 | 185.5 | 0.1 | 1.1 | 1 | PmuVarChr52859 | PmuLHCb13 |
| 2.30E-79 | 267.8 | 0.1 | 3.00E-79 | 267.4 | 0.1 | 1.1 | 1 | Mdg_01g010380 | MdgLhcb1 |
| 6.50E-72 | 243.4 | 0.1 | 8.30E-72 | 243 | 0.1 | 1.1 | 1 | Mdg_01g013510 | MdgLhcb2 |
| 5.70E-83 | 279.6 | 0.3 | 7.20E-83 | 279.3 | 0.3 | 1 | 1 | Mdg_02g008280 | MdgLhcb3 |
| 7.20E-100 | 335 | 0.6 | 8.50E-100 | 334.7 | 0.6 | 1 | 1 | Mdg_03g019460 | MdgLhcb4 |
| 4.60E-84 | 283.2 | 0 | 5.30E-84 | 283 | 0 | 1 | 1 | Mdg_04g013420 | MdgLhcb5 |
| 7.60E-91 | 305.4 | 0 | 8.80E-91 | 305.2 | 0 | 1 | 1 | Mdg_05g000180 | MdgLhcb6 |
| 7.60E-91 | 305.4 | 0 | 8.80E-91 | 305.2 | 0 | 1 | 1 | Mdg_05g000330 | MdgLhcb7 |
| 7.30E-109 | 364.4 | 0.1 | 8.30E-109 | 364.3 | 0.1 | 1 | 1 | Mdg_05g026060 | MdgLhcb8 |
| 7.20E-109 | 364.5 | 0.1 | 8.20E-109 | 364.3 | 0.1 | 1 | 1 | Mdg_05g026070 | MdgLhcb9 |
| 4.10E-95 | 319.4 | 0 | 4.80E-95 | 319.2 | 0 | 1 | 1 | Mdg_06g000210 | MdgLhcb10 |
| 4.00E-13 | 50.7 | 0 | 5.50E-13 | 50.3 | 0 | 1.1 | 1 | Mdg_06g001410 | MdgLhcb11 |
| 3.50E-80 | 270.5 | 0.1 | 4.10E-80 | 270.2 | 0.1 | 1 | 1 | Mdg_06g017660 | MdgLhcb12 |
| 7.20E-80 | 269.4 | 0.1 | 8.70E-80 | 269.2 | 0.1 | 1 | 1 | Mdg_07g015550 | MdgLhcb13 |
| 2.80E-23 | 84 | 0 | 3.30E-23 | 83.8 | 0 | 1.1 | 1 | Mdg_07g018460 | MdgLhcb14 |
| 4.70E-26 | 93.1 | 0 | 6.70E-26 | 92.6 | 0 | 1.2 | 1 | Mdg_07g019020 | MdgLhcb15 |
| 8.20E-17 | 62.8 | 0.1 | 2.20E-11 | 45 | 0 | 2 | 2 | Mdg_08g009080 | MdgLhcb16 |
| 3.00E-77 | 260.9 | 0.8 | 3.60E-77 | 260.6 | 0.8 | 1 | 1 | Mdg_08g019510 | MdgLhcb17 |
| 1.70E-105 | 353.4 | 0 | 2.40E-105 | 352.9 | 0 | 1.2 | 1 | Mdg_09g010860 | MdgLhcb18 |
| 6.70E-60 | 204 | 0.1 | 7.90E-60 | 203.8 | 0.1 | 1.1 | 1 | Mdg_09g011820 | MdgLhcb19 |
| 8.80E-38 | 131.5 | 0 | 1.00E-37 | 131.3 | 0 | 1 | 1 | Mdg_09g013140 | MdgLhcb20 |
| 2.00E-69 | 235.2 | 0.7 | 9.70E-56 | 190.4 | 0.1 | 2 | 2 | Mdg_09g024800 | MdgLhcb21 |
| 1.90E-90 | 304.1 | 0 | 2.20E-90 | 303.9 | 0 | 1 | 1 | Mdg_10g000060 | MdgLhcb22 |
| 1.70E-27 | 97.9 | 0 | 2.40E-27 | 97.4 | 0 | 1.2 | 1 | Mdg_11g021290 | MdgLhcb23 |
| 8.50E-100 | 334.7 | 0.4 | 1.00E-99 | 334.5 | 0.4 | 1 | 1 | Mdg_11g021420 | MdgLhcb24 |
| 1.20E-76 | 258.9 | 0 | 9.60E-73 | 246.1 | 0 | 2 | 1 | Mdg_12g015260 | MdgLhcb25 |
| 3.40E-85 | 286.9 | 0 | 4.90E-85 | 286.4 | 0 | 1.1 | 1 | Mdg_13g010190 | MdgLhcb26 |
| 1.10E-80 | 272.1 | 0.1 | 1.30E-80 | 271.9 | 0.1 | 1 | 1 | Mdg_14g018830 | MdgLhcb27 |
| 2.10E-88 | 297.4 | 0 | 2.30E-88 | 297.3 | 0 | 1 | 1 | Mdg_15g007840 | MdgLhcb28 |
| 3.40E-76 | 257.4 | 1.1 | 4.20E-76 | 257.1 | 1.1 | 1 | 1 | Mdg_15g036690 | MdgLhcb29 |
| 4.10E-85 | 286.6 | 0.1 | 5.30E-85 | 286.3 | 0.1 | 1 | 1 | Mdg_16g010720 | MdgLhcb30 |
| 4.60E-72 | 243.9 | 1.9 | 5.10E-54 | 184.7 | 0 | 2 | 2 | Mdg_17g010750 | MdgLhcb31 |
| 3.80E-105 | 352.2 | 0.1 | 5.70E-105 | 351.7 | 0.1 | 1.2 | 1 | Mdg_17g011070 | MdgLhcb32 |
| 7.90E-60 | 203.8 | 0.1 | 9.40E-60 | 203.5 | 0.1 | 1.1 | 1 | Mdg_17g011940 | MdgLhcb33 |
| 8.90E-110 | 367.4 | 0.1 | 1.10E-109 | 367.2 | 0.1 | 1 | 1 | Mdg_17g013070 | MdgLhcb34 |
| 5.50E-69 | 233.8 | 0.7 | 3.60E-55 | 188.5 | 0.1 | 2 | 2 | Mdg_17g025470 | MdgLhcb35 |
| 2.40E-111 | 372.4 | 0.1 | 2.80E-111 | 372.2 | 0.1 | 1 | 1 | PruarM.1G399400 | PruarMLhcb1 |
| 3.30E-111 | 372 | 0.1 | 3.80E-111 | 371.7 | 0.1 | 1 | 1 | PruarM.1G634500 | PruarMLhcb2 |
| 9.30E-111 | 370.5 | 0.1 | 1.10E-110 | 370.2 | 0.1 | 1.1 | 1 | PruarM.1G772300 | PruarMLhcb3 |
| 6.70E-110 | 367.7 | 0.1 | 8.10E-110 | 367.4 | 0.1 | 1 | 1 | PruarM.2G417000 | PruarMLhcb4 |
| 1.00E-108 | 363.8 | 0.1 | 1.20E-108 | 363.5 | 0.1 | 1 | 1 | PruarM.3G005300 | PruarMLhcb5 |
| 1.90E-108 | 362.9 | 0.1 | 2.30E-108 | 362.7 | 0.1 | 1 | 1 | PruarM.3G264300 | PruarMLhcb6 |
| 8.00E-106 | 354.3 | 0.1 | 9.30E-106 | 354.1 | 0.1 | 1 | 1 | PruarM.3G264400 | PruarMLhcb7 |
| 2.90E-100 | 336.1 | 0.5 | 3.40E-100 | 335.8 | 0.5 | 1 | 1 | PruarM.3G264400 | PruarMLhcb8 |
| 6.90E-96 | 321.8 | 0.4 | 1.00E-61 | 209.8 | 0.1 | 2 | 2 | PruarM.3G264500 | PruarMLhcb9 |
| 1.90E-91 | 307.2 | 0 | 2.30E-91 | 307 | 0 | 1 | 1 | PruarM.3G292200 | PruarMLhcb10 |
| 1.30E-89 | 301.2 | 0 | 1.50E-89 | 301 | 0 | 1 | 1 | PruarM.3G301500 | PruarMLhcb11 |
| 7.40E-84 | 282.3 | 0.2 | 8.80E-84 | 282.1 | 0.2 | 1 | 1 | PruarM.4G084000 | PruarMLhcb12 |
| 4.90E-82 | 276.4 | 0.1 | 5.50E-82 | 276.2 | 0.1 | 1 | 1 | PruarM.4G084100 | PruarMLhcb13 |
| 9.90E-81 | 272.1 | 0 | 1.50E-80 | 271.5 | 0 | 1.2 | 1 | PruarM.4G230600 | PruarMLhcb14 |
| 1.70E-79 | 268.1 | 0.1 | 2.00E-79 | 267.8 | 0.1 | 1 | 1 | PruarM.5G005900 | PruarMLhcb15 |
| 6.00E-76 | 256.4 | 1.3 | 7.40E-76 | 256.1 | 1.3 | 1 | 1 | PruarM.5G258000 | PruarMLhcb16 |
| 9.30E-75 | 252.5 | 0.3 | 3.60E-37 | 129.4 | 0 | 2.1 | 2 | PruarM.6G178800 | PruarMLhcb17 |
| 7.40E-71 | 239.7 | 0.1 | 9.90E-71 | 239.3 | 0.1 | 1.1 | 1 | PruarM.7G309200 | PruarMLhcb18 |
| 2.20E-57 | 195.6 | 0.1 | 2.80E-57 | 195.3 | 0.1 | 1.1 | 1 | PruarM.8G002400 | PruarMLhcb19 |
| 9.60E-28 | 98.5 | 0 | 1.30E-27 | 98 | 0 | 1.1 | 1 | PruarM.8G225500 | PruarMLhcb20 |
| 3.20E-111 | 371.3 | 0.1 | 3.70E-111 | 371.1 | 0.1 | 1 | 1 | evm.model.Chr1.1506 | P.saLhcb1 |
| 4.50E-110 | 367.5 | 0.1 | 5.50E-110 | 367.2 | 0.1 | 1 | 1 | evm.model.Chr1.3579.1 | P.saLhcb2 |
| 1.20E-99 | 333.3 | 0.5 | 1.60E-99 | 333 | 0.5 | 1.1 | 1 | evm.model.Chr1.373 | P.saLhcb3 |
| 1.20E-91 | 307.2 | 0 | 1.40E-91 | 307 | 0 | 1 | 1 | evm.model.Chr2.2463 | P.saLhcb4 |
| 1.60E-90 | 303.5 | 0 | 1.80E-90 | 303.3 | 0 | 1 | 1 | evm.model.Chr3.1865 | P.saLhcb5 |
| 2.20E-85 | 286.6 | 0 | 3.00E-85 | 286.2 | 0 | 1.1 | 1 | evm.model.Chr3.1866 | P.saLhcb6 |
| 6.50E-85 | 285.1 | 0.1 | 7.70E-85 | 284.8 | 0.1 | 1 | 1 | evm.model.Chr3.68 | P.saLhcb7 |
| 2.60E-84 | 283.1 | 0 | 3.10E-84 | 282.9 | 0 | 1 | 1 | evm.model.Chr4.1779 | P.saLhcb8 |
| 3.30E-84 | 282.8 | 0.2 | 3.90E-84 | 282.6 | 0.2 | 1 | 1 | evm.model.Chr5.2581 | P.saLhcb9 |
| 3.60E-82 | 276.1 | 0.1 | 4.10E-82 | 275.9 | 0.1 | 1 | 1 | evm.model.Chr5.503 | P.saLhcb10 |
| 9.40E-80 | 268.2 | 0.1 | 1.20E-79 | 267.9 | 0.1 | 1 | 1 | evm.model.Chr6.2772 | P.saLhcb11 |
| 5.50E-76 | 255.8 | 1.2 | 7.20E-76 | 255.4 | 1.2 | 1 | 1 | evm.model.Chr6.47 | P.saLhcb12 |
| 1.00E-72 | 245.1 | 0 | 1.20E-72 | 244.8 | 0 | 1.1 | 1 | evm.model.Chr7.2311 | P.saLhcb13 |
| 1.80E-48 | 165.7 | 1.2 | 1.60E-34 | 120 | 0.2 | 2 | 2 | evm.model.Chr8.1557 | P.saLhcb14 |
| 1.60E-44 | 152.8 | 0.1 | 1.90E-44 | 152.5 | 0.1 | 1 | 1 | evm.model.Chr8.16 | P.saLhcb15 |
| 1.40E-110 | 369.6 | 0 | 1.70E-110 | 369.3 | 0 | 1 | 1 | Ro01_G11269 | RoLhcb1 |
| 2.90E-110 | 368.6 | 0.1 | 3.60E-110 | 368.3 | 0.1 | 1.1 | 1 | Ro01_G29601 | RoLhcb2 |
| 4.20E-110 | 368.1 | 0.1 | 5.10E-110 | 367.8 | 0.1 | 1 | 1 | Ro02_G34905 | RoLhcb3 |
| 1.10E-109 | 366.7 | 0.1 | 1.30E-109 | 366.4 | 0.1 | 1 | 1 | Ro03_G13421 | RoLhcb4 |
| 1.80E-107 | 359.5 | 0.1 | 2.10E-107 | 359.2 | 0.1 | 1 | 1 | Ro03_G13424 | RoLhcb5 |
| 2.50E-107 | 359 | 0.1 | 3.10E-107 | 358.7 | 0.1 | 1 | 1 | Ro03_G15685 | RoLhcb6 |
| 1.20E-106 | 356.8 | 0.1 | 1.60E-106 | 356.3 | 0.1 | 1.1 | 1 | Ro03_G22595 | RoLhcb7 |
| 4.40E-106 | 354.9 | 0.1 | 5.30E-106 | 354.6 | 0.1 | 1 | 1 | Ro03_G33066 | RoLhcb8 |
| 1.70E-99 | 333.3 | 0.2 | 1.90E-99 | 333.2 | 0.2 | 1 | 1 | Ro04_G07134 | RoLhcb9 |
| 2.40E-92 | 309.9 | 0 | 2.80E-92 | 309.6 | 0 | 1 | 1 | Ro05_G03264 | RoLhcb10 |
| 1.90E-90 | 303.6 | 0 | 2.30E-90 | 303.4 | 0 | 1 | 1 | Ro05_G13866 | RoLhcb11 |
| 3.10E-83 | 280 | 0.2 | 3.60E-83 | 279.8 | 0.2 | 1 | 1 | Ro06_G08454 | RoLhcb12 |
| 9.20E-82 | 275.2 | 0.1 | 1.10E-81 | 275 | 0.1 | 1 | 1 | Ro06_G09967 | RoLhcb13 |
| 1.70E-80 | 271.1 | 0.1 | 2.00E-80 | 270.8 | 0.1 | 1 | 1 | Ro06_G14405 | RoLhcb14 |
| 2.20E-79 | 267.4 | 0.1 | 2.80E-79 | 267 | 0.1 | 1.1 | 1 | Ro06_G17315 | RoLhcb15 |
| 5.20E-79 | 266.2 | 0.1 | 6.50E-79 | 265.9 | 0.1 | 1 | 1 | Ro06_G20151 | RoLhcb16 |
| 9.70E-70 | 235.8 | 0 | 1.30E-69 | 235.4 | 0 | 1 | 1 | Ro06_G28525 | RoLhcb17 |
| 4.70E-37 | 128.7 | 0.2 | 7.80E-37 | 128 | 0.2 | 1.2 | 1 | Ro07_G07846 | RoLhcb18 |
| 1.70E-33 | 117.1 | 0 | 2.40E-33 | 116.5 | 0 | 1.1 | 1 | Ro07_G17114 | RoLhcb19 |
| 1.90E-171 | 569.1 | 0.2 | 1.50E-110 | 369.6 | 0.1 | 2 | 2 | Pp01G028600.1 | PpLhcb1 |
| 9.00E-106 | 354 | 0.1 | 1.10E-105 | 353.7 | 0.1 | 1 | 1 | Pp01G049950.1 | PpLhcb2 |
| 2.50E-100 | 336.1 | 0.5 | 3.00E-100 | 335.9 | 0.5 | 1 | 1 | Pp01G049950.2 | PpLhcb3 |
| 7.00E-100 | 334.7 | 1.3 | 2.50E-99 | 332.9 | 1.3 | 1.7 | 1 | Pp01G061790.1 | PpLhcb4 |
| 7.00E-100 | 334.7 | 1.3 | 2.50E-99 | 332.9 | 1.3 | 1.7 | 1 | Pp02G031400.1 | PpLhcb5 |
| 1.70E-91 | 307.2 | 0 | 2.00E-91 | 307 | 0 | 1 | 1 | Pp03G000410.1 | PpLhcb6 |
| 9.00E-91 | 304.8 | 0 | 1.00E-90 | 304.6 | 0 | 1 | 1 | Pp03G022800.1 | PpLhcb7 |
| 1.80E-85 | 287.4 | 0.1 | 2.20E-85 | 287.2 | 0.1 | 1.1 | 1 | Pp03G022810.1 | PpLhcb8 |
| 1.30E-84 | 284.6 | 0.1 | 1.50E-84 | 284.4 | 0.1 | 1 | 1 | Pp03G024590.1 | PpLhcb9 |
| 1.80E-84 | 284.2 | 0 | 2.10E-84 | 284 | 0 | 1 | 1 | Pp03G025520.1 | PpLhcb10 |
| 4.70E-84 | 282.8 | 0.2 | 5.50E-84 | 282.6 | 0.2 | 1 | 1 | Pp04G007050.1 | PpLhcb11 |
| 1.50E-83 | 281.1 | 1.8 | 2.90E-42 | 145.9 | 0.2 | 2 | 2 | Pp04G007060.1 | PpLhcb12 |
| 3.20E-82 | 276.8 | 0.1 | 3.60E-82 | 276.6 | 0.1 | 1 | 1 | Pp04G018100.1 | PpLhcb13 |
| 3.70E-80 | 270 | 0.1 | 4.50E-80 | 269.7 | 0.1 | 1 | 1 | Pp05G004780.1 | PpLhcb14 |
| 1.40E-77 | 261.5 | 0 | 1.90E-77 | 261.2 | 0 | 1.1 | 1 | Pp05G025710.1 | PpLhcb15 |
| 2.10E-76 | 257.8 | 0.1 | 3.80E-76 | 256.9 | 0.1 | 1.4 | 1 | Pp05G025710.2 | PpLhcb16 |
| 5.20E-76 | 256.4 | 1.3 | 6.40E-76 | 256.1 | 1.3 | 1 | 1 | Pp06G008360.1 | PpLhcb17 |
| 2.60E-70 | 237.8 | 0.1 | 3.40E-70 | 237.4 | 0.1 | 1.1 | 1 | Pp07G007170.1 | PpLhcb18 |
| 2.70E-59 | 201.7 | 0.1 | 3.10E-59 | 201.5 | 0.1 | 1.1 | 1 | Pp07G007190.1 | PpLhcb19 |
| 7.00E-57 | 193.8 | 0.8 | 1.00E-31 | 111.3 | 0.1 | 2 | 2 | Pp08G000180.1 | PpLhcb20 |
| 6.90E-21 | 75.8 | 0.3 | 8.80E-19 | 68.9 | 0 | 2 | 2 | Pp08G017780.1 | PpLhcb21 |
| 5.20E-109 | 364.9 | 0.1 | 6.10E-109 | 364.7 | 0.1 | 1 | 1 | RcHm_v2.0_Chr1g0363641 | RcHmLhcb1 |
| 2.40E-108 | 362.7 | 0.1 | 2.90E-108 | 362.5 | 0.1 | 1 | 1 | RcHm_v2.0_Chr1g0370011 | RcHmLhcb2 |
| 7.30E-108 | 361.2 | 0.1 | 8.70E-108 | 360.9 | 0.1 | 1 | 1 | RcHm_v2.0_Chr2g0095461 | RcHmLhcb3 |
| 2.60E-106 | 356.1 | 0 | 3.00E-106 | 355.9 | 0 | 1 | 1 | RcHm_v2.0_Chr2g0138781 | RcHmLhcb4 |
| 1.20E-105 | 353.9 | 0.1 | 1.40E-105 | 353.7 | 0.1 | 1 | 1 | RcHm_v2.0_Chr2g0140601 | RcHmLhcb5 |
| 1.40E-105 | 353.7 | 0.1 | 1.60E-105 | 353.5 | 0.1 | 1 | 1 | RcHm_v2.0_Chr2g0140611 | RcHmLhcb6 |
| 6.10E-99 | 331.9 | 0.3 | 6.90E-99 | 331.8 | 0.3 | 1 | 1 | RcHm_v2.0_Chr2g0140621 | RcHmLhcb7 |
| 8.60E-92 | 308.5 | 0 | 1.00E-91 | 308.3 | 0 | 1 | 1 | RcHm_v2.0_Chr2g0140661 | RcHmLhcb8 |
| 9.70E-90 | 301.8 | 0 | 1.10E-89 | 301.6 | 0 | 1 | 1 | RcHm_v2.0_Chr2g0140671 | RcHmLhcb9 |
| 6.40E-88 | 295.8 | 3.3 | 2.20E-35 | 123.7 | 0.3 | 3.8 | 3 | RcHm_v2.0_Chr2g0151701 | RcHmLhcb10 |
| 1.00E-84 | 285.3 | 0 | 1.20E-84 | 285.1 | 0 | 1 | 1 | RcHm_v2.0_Chr2g0155141 | RcHmLhcb11 |
| 3.10E-84 | 283.7 | 0.1 | 3.80E-84 | 283.5 | 0.1 | 1.1 | 1 | RcHm_v2.0_Chr2g0156601 | RcHmLhcb12 |
| 5.60E-84 | 282.9 | 0.1 | 6.30E-84 | 282.7 | 0.1 | 1 | 1 | RcHm_v2.0_Chr2g0156611 | RcHmLhcb13 |
| 7.10E-83 | 279.3 | 0.3 | 8.20E-83 | 279.1 | 0.3 | 1 | 1 | RcHm_v2.0_Chr2g0156621 | RcHmLhcb14 |
| 2.00E-81 | 274.6 | 0.1 | 2.30E-81 | 274.3 | 0.1 | 1 | 1 | RcHm_v2.0_Chr3g0463241 | RcHmLhcb15 |
| 9.80E-81 | 272.3 | 1.1 | 3.30E-70 | 237.8 | 0.3 | 2 | 2 | RcHm_v2.0_Chr3g0486251 | RcHmLhcb16 |
| 2.50E-79 | 267.7 | 0.1 | 2.90E-79 | 267.5 | 0.1 | 1 | 1 | RcHm_v2.0_Chr4g0430241 | RcHmLhcb17 |
| 1.20E-76 | 258.9 | 1 | 1.40E-76 | 258.6 | 1 | 1 | 1 | RcHm_v2.0_Chr5g0013251 | RcHmLhcb18 |
| 1.60E-76 | 258.5 | 0.8 | 5.40E-55 | 188 | 0 | 2 | 2 | RcHm_v2.0_Chr5g0013261 | RcHmLhcb19 |
| 9.50E-71 | 239.6 | 0.1 | 1.00E-70 | 239.4 | 0.1 | 1 | 1 | RcHm_v2.0_Chr5g0028381 | RcHmLhcb20 |
| 1.90E-70 | 238.6 | 0.7 | 5.60E-34 | 119.1 | 0.2 | 2 | 2 | RcHm_v2.0_Chr5g0036051 | RcHmLhcb21 |
| 2.10E-69 | 235.1 | 0.1 | 2.90E-69 | 234.7 | 0.1 | 1 | 1 | RcHm_v2.0_Chr5g0048261 | RcHmLhcb22 |
| 6.40E-63 | 213.9 | 0.5 | 4.50E-60 | 204.6 | 0.5 | 2 | 1 | RcHm_v2.0_Chr5g0067271 | RcHmLhcb23 |
| 8.90E-61 | 206.9 | 0.1 | 1.10E-60 | 206.6 | 0.1 | 1.1 | 1 | RcHm_v2.0_Chr5g0067731 | RcHmLhcb24 |
| 4.20E-54 | 185 | 1.8 | 5.60E-35 | 122.4 | 0.3 | 2 | 2 | RcHm_v2.0_Chr5g0067741 | RcHmLhcb25 |
| 5.00E-40 | 138.9 | 0.1 | 2.10E-37 | 130.3 | 0 | 2 | 2 | RcHm_v2.0_Chr5g0067781 | RcHmLhcb26 |
| 1.10E-38 | 134.4 | 0 | 1.20E-38 | 134.4 | 0 | 1 | 1 | RcHm_v2.0_Chr5g0067851 | RcHmLhcb27 |
| 5.10E-37 | 129 | 0.2 | 5.90E-32 | 112.5 | 0.1 | 2 | 2 | RcHm_v2.0_Chr5g0067871 | RcHmLhcb28 |
| 4.30E-33 | 116.2 | 0.1 | 4.90E-33 | 116 | 0.1 | 1 | 1 | RcHm_v2.0_Chr5g0067881 | RcHmLhcb29 |
| 1.70E-24 | 88 | 0 | 2.00E-24 | 87.8 | 0 | 1 | 1 | RcHm_v2.0_Chr6g0307891 | RcHmLhcb30 |
| 3.60E-24 | 86.9 | 0 | 3.80E-24 | 86.8 | 0 | 1 | 1 | RcHm_v2.0_Chr7g0181611 | RcHmLhcb31 |
| 5.00E-22 | 79.9 | 0 | 8.20E-22 | 79.2 | 0 | 1.3 | 1 | RcHm_v2.0_Chr7g0217391 | RcHmLhcb32 |
| 6.00E-13 | 50.2 | 0 | 8.50E-13 | 49.7 | 0 | 1.2 | 1 | RcHm_v2.0_Chr7g0226291 | RcHmLhcb33 |
