# Supplementary material for: Comparative genomics analysis provides insights into evolution and stress responses of Lhcb genes in Rosaceae fruit crops
Source: BMC Plant Biol. 2023 Oct 11;23:484. doi: 10.1186/s12870-023-04438-x (PMC10566169; doi:10.1186/s12870-023-04438-x)
Supplement: Supplementary file 5 — Additional file 5: Table S1-S6. [file 12870_2023_4438_MOESM5_ESM.zip › Supplemental Tables/Table S2.pdf]

Table S2 Number of cis-components in *PbrLHC*

gene_id	renamed	AAGAA	ARE	TCA-ele	A-box	AuxRR-cc	DRE core	GARE-mc	LTR	MBS	MRE	P-box	STRE	TC-rich r	TGACG-n	TGA-elem	CGTCA-n	W box	WRE3	GATA-mc	G-box	Box 4	TCT-motif	ATCT-motif	CAT-box	CCGTCC	GCN4_mc	O2-site	circadian	MYB
Pbr00087	*PbrLhcb23*		4										1		2		2	1			5		1		2			1		7
Pbr00168	*PbrLhcb11*		4		1	1				1			1		1	1	1	1		1	4	2	1	2		1	2			7
Pbr00239	*PbrLhcb27*	1	2	1	1			5	3	1		1		1		2		1	2	1	2	1	1	2	1	1		1	3	3
Pbr00239	*PbrLhcb28*	2								2	1		1			2			1	3	5	2		1	1					3
Pbr00239	*PbrLhcb29*		1						1	1		2	1						1	1			3		1			2	1	14
Pbr00428	*PbrLhcb9*	1	4				1	3					1		4		4		2		4	2		1	2					16
Pbr00526	*PbrLhcb13*	1	1					1		1					3	1	3	2			10		2		1			1		21
Pbr00729	*PbrLhcb21*	1	4	1						2			2		2		2	2	1	3	9	1	2		1		1	1		5
Pbr00805	*PbrLhcb5*	1	3						1	1	1		2		2		2			2	4	3	2							12
Pbr00860	*PbrLhcb25*			1									2					1		1	2	3					1			3
Pbr00951	*PbrLhcb20*	1	2						1		1	1	1		2	2	2		3	1	1	1		1	1					1
Pbr01089	*PbrLhcb8*		2				2			1	2		4	1	5		5	1	1	2	7				1					9
Pbr01142	*PbrLhcb6*	1	2									1	3	1	1		1	3	1	1	7				1		1	1	1	15
Pbr01279	*PbrLhcb1*	1	3	1				1	1				5	1	2		2	1		2	3	1			2					10
Pbr01512	*PbrLhcb7*		3							1			2		4		4		3		3	1								10
Pbr01516	*PbrLhcb24*	1	5						1	1			2	2	2		2	1	2	1	4	1	1					2		11
Pbr01909	*PbrLhcb10*		4			1				1			1		1	1	1	1		2	4	3	1	1			1			13
Pbr01963	*PbrLhcb22*	1	4		1				1			1	2		3		3	1	1		3		1		2	1				11
Pbr02165	*PbrLhcb4*	1		1				1	2	2	1	1	5		3		3		1	2		3			1					8
Pbr02204	*PbrLhcb19*	1	2		1							1	3		3	1	3		2	1	11	2	2	1	4	1		1		10
Pbr02391	*PbrLhcb18*	1	3							1			5			2		1	3		3	1		1	1			1	2	12
Pbr02483	*PbrLhcb3*	1	1		1					2		1	2		3		3		2		6	1				1		3		11
Pbr02773	*PbrLhcb2*		1									2	1		1		1	2	1	1	2	2	1							13
Pbr02964	*PbrLhcb12*	1	6	4									1					1		1	3	2			1		1			13
Pbr03325	*PbrLhcb16*	8	2								1		5						3	1	2	2			2		1	3		9
Pbr03630	*PbrLhcb26*		2	1				1		1			1		1		1	1		1	5		1		1			1		2
Pbr03791	*PbrLhcb17*	3				1			1			2	4		3		3	1		1	3	1	1	3	1			1		14
Pbr03955	*PbrLhcb15*	1	2			1			1	3		1						1		1	6	1		1	3			1		15
Pbr03955	*PbrLhcb14*	1	2		1	1			1	3	1	1						1		1	7	1		1	3	1		1		9
Pbr04026	*PbrLhc1*	3							1			2	4	2				1		2	8		1	1	1			2		12
Pbr00538	*PbrLhc2*	1	2		1		1		1		1		4		4	1	4		1		4	2				1				1
Pbr01375	*PbrLhc3*	1										2	4	2	1	1	1			2	8		2	1	1			2		9
Pbr01409	*PbrLhc4*		1		1		1		1	2			1		2	1	2	1	1		6	3	2		3	1	1			12
Pbr01695	*PbrLhc5*	1	1	1				1	1	1			4	1	3	1	3				3	1	2		2			1		12
Pbr01927	*PbrLhc6*		1	1		1			3	1			1		5		5			1	5	2	1		1			1		8
Pbr02282	*PbrLhc7*		2	1					2	1	1	1	3			1					1									9
Pbr02748	*PbrLhc8*		3						2		1		1	1	3	1	3	1	1	1	8	3	1					2		9
