# Supplementary material for: Comparative genomics analysis provides insights into evolution and stress responses of Lhcb genes in Rosaceae fruit crops
Source: BMC Plant Biol. 2023 Oct 11;23:484. doi: 10.1186/s12870-023-04438-x (PMC10566169; doi:10.1186/s12870-023-04438-x)
Supplement: Supplementary file 5 — Additional file 5: Table S1-S6. [file 12870_2023_4438_MOESM5_ESM.zip › Supplemental Tables/Table S3.docx]

| Table S3 The ks value of the duplicate gene pair | | | | |
| --- | --- | --- | --- | --- |
| Gene1 | Gene2 | Ka | Ks | Ka_Ks |
| Pbr023911.1 | Pbr022044.2 | 0.016139296 | 0.2138842 | 0.0754581 |
| Pbr009517.1 | Pbr021654.1 | 0.163335061 | 1.1118166 | 0.1469083 |
| Pbr009517.1 | Pbr010895.1 | 0.032683639 | 0.2273806 | 0.1437398 |
| Pbr039555.1 | Pbr002394.1 | 0.090832206 | 1.2607927 | 0.0720437 |
| Pbr039555.1 | Pbr029644.1 | 0.059490632 | 1.790088 | 0.0332334 |
| Pbr033256.1 | Pbr024832.1 | 0.005025846 | 0.2143602 | 0.0234458 |
| Pbr037913.1 | Pbr021654.1 | 0.009370242 | 0.240744 | 0.038922 |
| Pbr007291.1 | Pbr011422.1 | 0.017452795 | 0.2490159 | 0.0700871 |
| Pbr000879.2 | Pbr004280.1 | 0.036663536 | 0.2317477 | 0.1582045 |
| Pbr002394.1 | Pbr029644.1 | 0.039495481 | 0.6255098 | 0.0631413 |
| Pbr036302.1 | Pbr005261.1 | 0.012575396 | 0.1967409 | 0.0639186 |
| Pbr008607.1 | Pbr001687.1 | 0 | 0.1709491 | 0 |
| Pbr008607.1 | Pbr019098.1 | 0 | 0.1582571 | 0 |
| Pbr021654.1 | Pbr010895.1 | 0.249821901 | 1.5838796 | 0.1577278 |
| Pbr019098.1 | Pbr001687.1 | 0 | 0.020691 | 0 |
| FvH4_2g06100.1 | FvH4_2g34470.1 | 0.233658322 | 1.8729768 | 0.1247524 |
| FvH4_3g06110.1 | FvH4_6g38390.1 | 0.173000531 | 1.5376284 | 0.1125113 |
| FvH4_6g11540.1 | FvH4_7g24350.1 | 0.259641941 | 1.54 | 1.63 |
| Mdg_01g013510-mRNA1 | Mdg_12g015260-mRNA1 | 0.174525351 | 1.2460185 | 0.1400664 |
| Mdg_01g013510-mRNA1 | Mdg_04g013420-mRNA1 | 0.172540822 | 1.3830956 | 0.1247497 |
| Mdg_01g010380-mRNA1 | Mdg_07g015550-mRNA1 | 0.00891541 | 0.199677 | 0.0446492 |
| Mdg_01g013510-mRNA1 | Mdg_07g019020-mRNA1 | 0.153928548 | 0.3485214 | 0.4416616 |
| Mdg_10g000060-mRNA1 | Mdg_05g000330-mRNA1 | 0.013937683 | 0.1921768 | 0.0725253 |
| Mdg_10g000060-mRNA1 | Mdg_05g000180-mRNA1 | 0.013937683 | 0.1921768 | 0.0725253 |
| Mdg_11g021420-mRNA1 | Mdg_03g019460-mRNA1 | 0.00502164 | 0.2150047 | 0.023356 |
| Mdg_12g015260-mRNA1 | Mdg_04g013420-mRNA1 | 0.014845846 | 0.2686025 | 0.0552707 |
| Mdg_12g015260-mRNA1 | Mdg_07g019020-mRNA1 | 0.173190947 | 1.2013285 | 0.1441662 |
| Mdg_13g010190-mRNA1 | Mdg_16g010720-mRNA1 | 0.019211296 | 0.1944522 | 0.098797 |
| Mdg_14g018830-mRNA1 | Mdg_06g017660-mRNA1 | 0.028118683 | 0.2186728 | 0.1285879 |
| Mdg_15g036690-mRNA1 | Mdg_08g019510-mRNA1 | 0.038706918 | 0.2213279 | 0.174885 |
| Mdg_15g007840-mRNA1 | Mdg_08g009080-mRNA1 | 0.249273266 | 0.6139675 | 0.406004 |
| Mdg_17g013070-mRNA1 | Mdg_05g026060-mRNA1 | 0.062336908 | 1.0981643 | 0.0567646 |
| Mdg_17g011070-mRNA1 | Mdg_09g010860-mRNA1 | 0.021571289 | 0.2086576 | 0.1033813 |
| Mdg_17g011940-mRNA1 | Mdg_09g011820-mRNA1 | 0.012920912 | 0.2782188 | 0.0464415 |
| Mdg_17g013070-mRNA1 | Mdg_09g013140-mRNA1 | 0.065309303 | 0.8204047 | 0.0796062 |
| Mdg_17g025470-mRNA1 | Mdg_09g024800-mRNA1 | 0.002265007 | 0.1946334 | 0.0116373 |
| Mdg_04g013420-mRNA1 | Mdg_07g019020-mRNA1 | 0.217068636 | 1.4087999 | 0.1540805 |
| Mdg_05g026060-mRNA1 | Mdg_09g013140-mRNA1 | 0.101749399 | 1.2322607 | 0.0825713 |
| evm.model.Chr2.2463 | evm.model.Chr6.2772 | 0.141818348 | 1.4550823 | 0.0974641 |
| evm.model.Chr5.2581 | evm.model.Chr6.47 | 0.03200047 | 0.0216874 | 1.4755347 |
| evm.model.Chr1.3733 | evm.model.Chr3.3238.1 | 0.386507349 | 1.6666577 | 0.2319057 |
| evm.model.Chr3.689 | evm.model.Chr7.2645 | 0.443084158 | 1.643629 | 0.2695767 |
| evm.model.Chr3.688 | evm.model.Chr7.2646.1 | 0.51659605 | 2.1873648 | 0.2361728 |
| evm.model.Chr3.687 | evm.model.Chr7.2647 | 0.229077526 |  |  |
| evm.model.Chr3.683.2 | evm.model.Chr7.2649 | 0.174519674 | 1.291691 | 0.1351095 |
| evm.model.Chr3.682 | evm.model.Chr7.2652 | 0.480628828 |  |  |
| evm.model.Chr8.159_evm.model.Chr8.160 | evm.model.Chr8.226.1 | 9.43E-04 | 0 |  |
| evm.model.Chr8.161 | evm.model.Chr8.227.1 | 8.35E-04 | 0 |  |
| evm.model.Chr8.162 | evm.model.Chr8.229 | 0.177542567 | 0.2313248 | 0.7675035 |
| evm.model.Chr8.163 | evm.model.Chr8.230 | 0 | 0.0098483 | 0 |
| evm.model.Chr8.164 | evm.model.Chr8.231_evm.model.Chr8.233 | 0 | 0.0191215 | 0 |
| evm.model.Chr8.167 | evm.model.Chr8.234 | 0.001009082 | 0.0070923 | 0.1422795 |
| Pp01G053360.1 | Pp01G054060.1 | 0.323268531 | 0.818201 | 0.3950967 |
| Pp01G053540.1 | Pp01G053950.1 | 0.429153802 | 1.0264874 | 0.4180799 |
| Pp01G053740.1 | Pp01G053750.1 | 0.41665322 | 0.9596373 | 0.4341778 |
| PruarM.1G634500.t1.p1 | PruarM.8G205400.t1.p1 | 0.19420878 | 3.2308813 | 0.0601102 |
| PruarM.3G264300.t1.p1 | PruarM.4G084000.t1.p1 | 0.069452219 | 1.2375616 | 0.0561202 |
| mRNA:RcHm_v2.0_Chr1g0370011 | mRNA:RcHm_v2.0_Chr3g0463241 | 0.184194006 |  |  |
| mRNA:RcHm_v2.0_Chr2g0151701 | mRNA:RcHm_v2.0_Chr5g0013251 | 0.016533391 | 3.4860617 | 0.0047427 |
| Ro03_G13424 | Ro06_G28525 | 0.052597377 | 1.336686 | 0.0393491 |
| Ro06_G14405 | Ro07_G07846 | 0.317123955 | 1.9862004 | 0.1596636 |
