# Supplementary material for: Comparative genomics analysis provides insights into evolution and stress responses of Lhcb genes in Rosaceae fruit crops
Source: BMC Plant Biol. 2023 Oct 11;23:484. doi: 10.1186/s12870-023-04438-x (PMC10566169; doi:10.1186/s12870-023-04438-x)
Supplement: Supplementary file 5 — Additional file 5: Table S1-S6. [file 12870_2023_4438_MOESM5_ESM.zip › Supplemental Tables/Table S4.docx]

| Table S4 Design of primer for qRT-PCR | |
| --- | --- |
| Primer name | Primer sequence |
| Pbr015123 F | GAAGCGGTTGGAGGAAAGGA |
| Pbr015123 R | CTAGCATAGCCCAACGAGCA |
| Pbr021654 F | TTGGGCTGCAGAGGTTTAGG |
| Pbr021654 R | AGAAAGGCAGTGGTTGACCC |
| Pbr022044 F | TTTGATGAGCTGTGGCATCG |
| Pbr022044 R | CAAAGTCACCGGGGGCG |
| Pbr027732 F | GCTCTGGTTTGACCCCATGA |
| Pbr027732 R | AGAACCCTACGAAAGCGAGC |
| Pbr033613 F | CGGCGGTTTGTCCATTTCAG |
| Pbr033613 R | CATGCGGTCATTGGTATCTGC |
| Pbr037615 F | AATCCGATTCCGAAGGAGCC |
| Pbr037615 R | AAAGACGGGTACGACGTTTC |
| Pbr040964 F | AAAGACGGGTACGACGTTTC |
| Pbr040965 R | ATAACGGCAGCACAAAACGC |
| Tubulin F | TGGGCTTTGCTCCTCTTAC |
| Tubulin R | CCTTCGTGCTCATCTTACC |
