# Supplementary material for: Comparative genomics analysis provides insights into evolution and stress responses of Lhcb genes in Rosaceae fruit crops
Source: BMC Plant Biol. 2023 Oct 11;23:484. doi: 10.1186/s12870-023-04438-x (PMC10566169; doi:10.1186/s12870-023-04438-x)
Supplement: Supplementary file 5 — Additional file 5: Table S1-S6. [file 12870_2023_4438_MOESM5_ESM.zip › Supplemental Tables/Table S5.docx]

| Table S5 The renaming of a gene name | |
| --- | --- |
| Gene_id | renamed |
| Pbr000879.2 | PbrLhcb23 |
| Pbr001687.1 | PbrLhcb11 |
| Pbr002394.1 | PbrLhcb27 |
| Pbr002395.1 | PbrLhcb28 |
| Pbr002396.1 | PbrLhcb29 |
| Pbr004280.1 | PbrLhcb9 |
| Pbr005261.1 | PbrLhcb13 |
| Pbr007291.1 | PbrLhcb21 |
| Pbr008050.1 | PbrLhcb5 |
| Pbr008607.1 | PbrLhcb25 |
| Pbr009517.1 | PbrLhcb20 |
| Pbr010895.1 | PbrLhcb8 |
| Pbr011422.1 | PbrLhcb6 |
| Pbr012798.1 | PbrLhcb1 |
| Pbr015123.1 | PbrLhcb7 |
| Pbr015169.1 | PbrLhcb24 |
| Pbr019098.1 | PbrLhcb10 |
| Pbr019632.1 | PbrLhcb22 |
| Pbr021654.1 | PbrLhcb4 |
| Pbr022044.2 | PbrLhcb19 |
| Pbr023911.1 | PbrLhcb18 |
| Pbr024832.1 | PbrLhcb3 |
| Pbr027732.1 | PbrLhcb2 |
| Pbr029644.1 | PbrLhcb12 |
| Pbr033256.1 | PbrLhcb16 |
| Pbr036302.1 | PbrLhcb26 |
| Pbr037913.1 | PbrLhcb17 |
| Pbr039554.1 | PbrLhcb15 |
| Pbr039555.1 | PbrLhcb14 |
| Pbr040268.1 | PbrLhc1 |
| Pbr005387.1 | PbrLhc2 |
| Pbr013750.1 | PbrLhc3 |
| Pbr014098.1 | PbrLhc4 |
| Pbr016957.1 | PbrLhc5 |
| Pbr019270.1 | PbrLhc6 |
| Pbr022822.1 | PbrLhc7 |
| Pbr027481.1 | PbrLhc8 |
| AT1G15820.1 | AtLhcb1 |
| AT1G29910.1 | AtLhcb2 |
| AT1G29920.1 | AtLhcb3 |
| AT1G29930.1 | AtLhcb4 |
| AT1G76570.1 | AtLhcb5 |
| AT2G05070.1 | AtLhcb6 |
| AT2G05100.1 | AtLhcb7 |
| AT2G34420.1 | AtLhcb8 |
| AT2G34430.1 | AtLhcb9 |
| AT2G40100.1 | AtLhcb10 |
| AT2G40100.2 | AtLhcb11 |
| AT3G08940.1 | AtLhcb12 |
| AT3G08940.2 | AtLhcb13 |
| AT3G27690.1 | AtLhcb14 |
| AT4G10340.1 | AtLhcb15 |
| AT5G01530.1 | AtLhcb16 |
| AT5G54270.1 | AtLhcb17 |
| XP_021593162.1 | MeLhcb1 |
| XP_021606221.1 | MeLhcb2 |
| XP_021606220.1 | MeLhcb3 |
| XP_021612355.1 | MeLhcb4 |
| XP_021631645.1 | MeLhcb5 |
| XP_021629416.1 | MeLhcb6 |
| XP_021621616.1 | MeLhcb7 |
| XP_021615578.1 | MeLhcb8 |
| XP_021592964.1 | MeLhcb9 |
| XP_021617472.1 | MeLhcb10 |
| XP_021631529.1 | MeLhcb11 |
| XP_021625988.1 | MeLhcb12 |
| XP_021617482.1 | MeLhcb13 |
| XP_021606144.1 | MeLhcb14 |
| XP_021599890.1 | MeLhcb15 |
| XP_021599422.1 | MeLhcb16 |
| XP_021599266.1 | MeLhcb17 |
| XP_021593253.1 | MeLhcb18 |
| XP_021593252.1 | MeLhcb19 |
| XP_021593834.2 | MeLhcb20 |
| XP_021629158.1 | MeLhcb21 |
| XP_021629080.2 | MeLhcb22 |
| XP_021594741.1 | MeLhcb23 |
