# Supplementary material for: Comparative genomics analysis provides insights into evolution and stress responses of Lhcb genes in Rosaceae fruit crops
Source: BMC Plant Biol. 2023 Oct 11;23:484. doi: 10.1186/s12870-023-04438-x (PMC10566169; doi:10.1186/s12870-023-04438-x)
Supplement: Supplementary file 5 — Additional file 5: Table S1-S6. [file 12870_2023_4438_MOESM5_ESM.zip › Supplemental Tables/Table S6.docx]

| **Table S6** Hypothesized function of the Lhcb gene family in Rosaceae | | | | |
| --- | --- | --- | --- | --- |
| Super groups | Rosaceae | *A. thaliana* and  other plants | Functions | References |
| LhcbI | *FvLHCb14 PpLhcb12 RoLhcb4 RoLhcb5 RoLhcb8 PbrLhcb14 PbrLhcb15 MdgLhcb8 MdgLhcb9 FvLHCb3 FvLHCb4 RcHmLhcb19 RcHmLhcb18 PmuLHCb6 PruarMLhcb6 PruarMLhcb5 PpLhcb5 PpLhcb4 RoLhcb17 FvLHCb7 FvLHCb13 RcHmLhcb29 RcHmLhcb6 RoLhcb16 FvLHCb16 FvLHCb15 RcHmLhcb10 MdgLhcb30 RcHmLhcb23 PruarMLhcb9 PmuLHCb12 PbrLhcb12 PbrLhcb28 PbrLhcb27 MdgLhcb33 PbrLhcb29 PmuLHCb10 RoLhcb13 PpLhcb1 PruarMLhcb2 PsaLhcb6 PmuLHCb1 PruarMLhcb3 PmuLHCb11 MdgLhcb20 PsaLhcb7 PruarMLhcb4 MdgLhcb34 RcHmLhcb16 RoLhcb1 PbrLhcb11 PbrLhcb10 PbrLhcb25* | *AtLhcb9(LHCB1.4) AtLhcb8(LHCB1.5) AtLhcb2(LHCB1.2, CAB3) AtLhcb4(CAB1) AtLhcb3(LHCB1.1, CAB2)*  *MeLhcb9 MeLhcb8 MeLhcb7* | Regulate circaqqdian rhythm and light response | [1] |
| LhcbII | *RoLhcb15 MdgLhcb13 RcHmLhcb4 PbrLhcb26 MdgLhcb31 MdgLhcb17 PbrLhcb13 PruarMLhcb7 PpLhcb2 RcHmLhcb14 RcHmLhcb13 RcHmLhcb12 FvLHCb18 FvLHCb21 FvLHCb20 FvLHCb19* | *AtLhcb14(LHCB2.4) AtLhcb7(LHCB2,1) AtLhcb6(LHCB2.2) MeLhcb20 MeLhcb19* | Stress responses | [2] |
| LhcbIII | *FvLHCb6 RcHmLhcb21 RoLhcb7 PsaLhcb8 PpLhcb3 PruarMLhcb8 PmuLHCb8 MdgLhcb23 PbrLhcb16 MdgLhcb4 PbrLhcb3* | *MeLhcb10*  *AtLhcb17(LHCB3.1)* | Coordination of Chloroplast Development | [3] |
| LhcbIV | *FvLHCb24 RoLhcb14 MdgLhcb14 PbrLhcb8 PmuLHCb13 PbrLhcb20 RcHmLhcb2 RoLhcb18 MdgLhcb2 PruarMLhcb18 PpLhcb18 PmuLHCb2 PsaLhcb11 PpLhcb10 FvLHCb12 MdgLhcb24 RcHmLhcb15 PbrLhcb17 MdgLhcb5 PbrLhcb4*  *MdgLhcb15 MdgLhcb27 PbrLhcb22 RoLhcb3 RcHmLhcb30 FvLHCb2 PpLhcb16 PruarMLhcb14 PmuLHCb4 PsaLhcb1 PpLhcb9 MdgLhcb26 PbrLhcb21 RcHmLhcb31 FvLHCb9 RoLhcb10 MdgLhcb11 PbrLhcb6 PsaLhcb10 PpLhcb13 PruarMLhcb13 PmuLHCb16 PruarMLhcb17 RcHmLhcb17 FvLHCb8 RoLhcb9 PsaLhcb2 PbrLhcb24 PpLhcb8 MdgLhcb29 MdgLhcb25 PpLhcb21 RcHmLhcb3 FvLHCb1 RoLhcb2 MdgLhcb3 PbrLhcb2 PmuLHCb17 PpLhcb11 PsaLhcb13 PruarMLhcb12* | *AtLhcb12(LHCB4.2) AtLhcb11(LHCB4.2) AtLhcb10(LHCB4.2) MeLhcb17 AtLhcb16(LHCB4.1) AtLhcb13(LHCB4.2)*  *MeLhcb14 MeLhcb1*  *MeLhcb15*  *MeLhcb23 MeLhcb21 MeLhcb12 MeLhcb11 MeLhcb13* | Stress responses and Abscisic acid biosynthesis | [2,4,5] |
| LhcbV | *RoLhcb6 RcHmLhcb20 FvLHCb5 PpLhcb7 PsaLhcb15 PruarMLhcb11 PmuLHCb14 MdgLhcb21 PbrLhcb1 PbrLhcb5 MdgLhcb7 MdgLhcb6*  *FvLHCb22 RcHmLhcb9 PpLhcb20*  *PruarMLhcb20* | *MeLhcb3 MeLhcb5 MeLhcb4 AtLhcb15(LHCB5)* | Stress responses and Light responses | [5,6] |
| LhcbVI | *FvLHCb23 RoLhcb19 MdgLhcb12 PbrLhcb18 MdgLhcb1 PbrLhcb19 RcHmLhcb1 PpLhcb14 PmuLHCb15 PsaLhcb14 PruarMLhcb15*  *FvLHCb11 RcHmLhcb33 MdgLhcb28 PbrLhcb23 MdgLhcb16 PbrLhcb9 PpLhcb17 PmuLHCb3 PsaLhcb3 PruarMLhcb16* | *MeLhcb18 MeLhcb16*  *AtLhcb1(LHCB6, CP24)*  *MeLhcb2* | Growth and development | [7,8] |
| LhcbVII | *MdgLhcb10 PbrLhcb7 RoLhcb11 RcHmLhcb32 FvLHCb10 PsaLhcb9 PpLhcb15 PpLhcb6 PmuLHCb5 PsaLhcb12 PruarMLhcb10*  *RoLhcb12 RcHmLhcb11 FvLHCb17 PruarMLhcb19 PmuLHCb9 PpLhcb19 MdgLhcb32 MdgLhcb18* | *AtLhcb5(LHCB7) MeLhcb6 MeLhcb22* | Growth and development | [9] |

1.Kreslavski VD, Khudyakova AY, Strokina VV, Shirshikova GN, Pashkovskiy PP, Balakhnina TI, Kosobryukhov AA, Kuznetsov VV, Allakhverdiev SI: Impact of high irradiance and UV-B on the photosynthetic activity, pro-/antioxidant balance and expression of light-activated genes in Arabidopsis thaliana hy4 mutants grown under blue light. Plant Physiology and Biochemistry 2021, 167:153-162.

2. Lellis AD, Patrick RM, Mayberry LK, Lorence A, Campbell ZC, Roose JL, Frankel LK, Bricker TM, Hellmann HA, Mayberry RW *et al*: eIFiso4G Augments the Synthesis of Specific Plant Proteins Involved in Normal Chloroplast Function. Plant Physiology 2019.

3. Mekala NR, Suorsa M, Rantala M, Aro E-M, Tikkanen M: Plants Actively Avoid State Transitions upon Changes in Light Intensity: Role of Light-Harvesting Complex II Protein Dephosphorylation in High Light. Plant Physiology 2015, 168(2):721-734.

4. Robinson SJ, Parkin IAP: Differential SAGE analysis in Arabidopsis uncovers increased transcriptome complexity in response to low temperature. BMC Genomics 2008, 9(1):434.

5. Soitamo AJ, Piippo M, Allahverdiyeva Y, Battchikova N, Aro E-M: Light has a specific role in modulating Arabidopsis gene expression at low temperature. BMC Plant Biology 2008, 8(1):13.

6. Chen Y-E, Liu W-J, Su Y-Q, Cui J-M, Zhang Z-W, Yuan M, Zhang H-Y, Yuan S: Different response of photosystem II to short and long-term drought stress in Arabidopsis thaliana. Physiologia Plantarum 2016, 158(2):225-235.

7. Ilíková I, Ilík P, Opatíková M, Arshad R, Nosek L, Karlický V, Kučerová Z, Roudnický P, Pospíšil P, Lazár D *et al*: Towards spruce-type photosystem II: consequences of the loss of light-harvesting proteins LHCB3 and LHCB6 in Arabidopsis. Plant Physiology 2021, 187(4):2691-2715.

8. Cortleven A, Marg I, Yamburenko MV, Schlicke H, Hill K, Grimm B, Schaller GE, Schmülling T: Cytokinin Regulates the Etioplast-Chloroplast Transition through the Two-Component Signaling System and Activation of Chloroplast-Related Genes. Plant Physiology 2016, 172(1):464-478.

9. Klimmek F, Sjödin A, Noutsos C, Leister D, Jansson S: Abundantly and rarely expressed Lhc protein genes exhibit distinct regulation patterns in plants. Plant Physiology 2006, 140(3):793-804.
